# Regulation of pyroptosis by natural products in ulcerative colitis: mechanisms and therapeutic potential

**DOI:** 10.3389/fphar.2025.1573684

**Published:** 2025-04-09

**Authors:** Xiaobei Lu, Yapeng Sun, Zhaoyi Zhang, Zhigang Sun, Shaohui Wang, Erping Xu

**Affiliations:** ^1^ Traditional Chinese Medicine (Zhong Jing) School, Henan University of Chinese Medicine, Zhengzhou, China; ^2^ Department of Proctology, Third Affiliated Hospital of Henan University of Traditional Chinese Medicine, Zhengzhou, China; ^3^ Qingdao Academy of Chinese Medicinal Sciences, Shandong University of Traditional Chinese Medicine, Qingdao, China

**Keywords:** ulcerative colitis, pyroptosis, natural products, NLRP3, flavonoids, polyphenols

## Abstract

Ulcerative colitis (UC), a chronic inflammatory bowel disease, is driven by dysregulated immune responses and persistent intestinal inflammation. Pyroptosis, a caspase/gasdermin-mediated inflammatory cell death that exacerbates mucosal damage through excessive cytokine release and epithelial barrier disruption. Although pyroptosis is considered to be a key mechanism in the pathogenesis of UC, the systematic assessment of the role of natural products in targeting the pyroptosis pathway remains a critical research gap. The purpose of this review is to investigate the regulatory effects of natural products on pyroptosis in UC and elucidate the mechanisms of action and potential therapeutic effects. Key findings highlight polyphenols (e.g., resveratrol), flavonoids (e.g., Quercetin), and terpenoids as promising agents that inhibit NLRP3 inflammasome activation, suppress gasdermin D cleavage, and restore barrier integrity, thereby reducing pro-inflammatory cytokine release in preclinical UC models. Current evidence shows enhanced efficacy and safety when these compounds are combined with standard therapies, but clinical translation requires overcoming three key barriers: limited human trial data, uncharacterized polypharmacology, and suboptimal pharmacokinetics needing formulation refinement. Future research should prioritize standardized animal-to-human translational models, mechanistic studies on synergistic pathways, and rigorous clinical validation to harness the full potential of natural products in pyroptosis-targeted UC therapies.

## Graphical Abstract

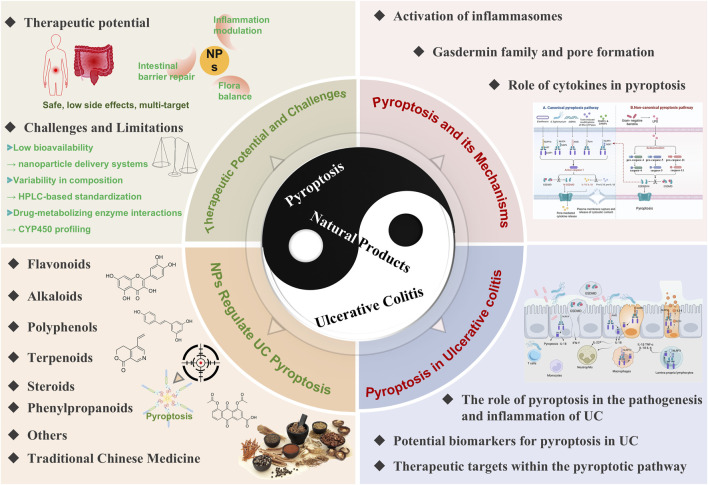

## 1 Introduction

Ulcerative colitis (UC) is a persistent inflammatory condition of the bowel distinguished by the ulceration and inflammation of the mucosal lining of the colon (Wangchuk et al., 2024). Recent trends in epidemiology indicate a concerning global rise in prevalence, with estimates suggesting that approximately 5 million people may be affected worldwide by 2023 (Le Berre et al., 2023). UC typically manifests with a range of symptoms including abdominal pain, diarrhea, rectal bleeding, and urgency, significantly impairing the quality of life for patients (Gros and Kaplan, 2023; Wangchuk et al., 2024). The disease often follows a relapsing-remitting course, wherein periods of exacerbation are interspersed with remission, making its management particularly challenging (Gajendran et al., 2019). The etiology of UC remains multifactorial, involving genetic predisposition, dysregulation of the immune response, and environmental factors, including diet and gut microbiota (Kazmi et al., 2024; Long et al., 2024a). Current therapeutic strategies aim to induce and maintain remission through the use of anti-inflammatory agents, immunosuppressants, and biologics (Liu et al., 2024). Although these current treatments can achieve some therapeutic effect, they are often accompanied by significant side effects (Zhao L. et al., 2024). Therefore, there is an urgent need for new, safer and more effective treatment strategies.

Recent research has begun to unravel the complex role of pyroptosis, a highly inflammatory form of programmed cell death, in the pathogenesis of UC (Zhen and Zhang, 2019). Pyroptosis is characterized by cell swelling, membrane rupture, and the release of pro-inflammatory cytokines, which can exacerbate tissue damage and inflammation. This process is primarily mediated by inflammasomes, multi-protein complexes that detect pathogenic stimuli and activate caspase-1, leading to the cleavage of gasdermin D (GSDMD) and subsequent cell lysis (Vasudevan et al., 2023). In the context of UC, pyroptosis contributes to the inflammatory response and mucosal injury, highlighting its potential as a therapeutic target.

Natural products have long been a cornerstone of medicine, with many modern pharmaceuticals deriving from compounds found in plants, fungi, and other natural sources. The importance of natural products as an important source for the development of new drugs is highlighted (Ekiert and Szopa, 2020; Zhang L. et al., 2020). As an example, salicylic acid, which is obtained from willow bark, was instrumental in paving the way for aspirin’s creation (Peng B. et al., 2024). Natural products are of interest due to their wide range of biological activities and studies have found that they are often able to enhance their therapeutic potential by modulating multiple targets and pathways (Abdulhakeem Mansour Alhasbary et al., 2025). Recent studies have demonstrated that specific natural compounds can modulate inflammatory responses by influencing pyroptosis pathways, indicating the potential for these compounds to exert their anti-inflammatory effects through a novel mechanism (Zhang et al., 2023; Sun J. et al., 2024). The interplay between natural products and pyroptosis in UC represents a promising area for further research and therapeutic development. By understanding how these compounds modulate the pyroptosis process may lead us to novel approaches to mitigate the inflammatory process in UC. In summary, the close association between pyroptosis and UC, and the therapeutic promise of natural products, make this a new area of widespread interest. Therefore, this review summarizes the role of pyroptosis in UC, and highlights the potential pharmacological mechanisms of natural products targeting pyroptosis in the treatment of UC, and discusses their therapeutic potential in UC to pave the way for future research and clinical application.

## 2 Literature search strategy

A systematic literature search spanning the decade from 2014 to 2024, was conducted across ScienceDirect, PubMed, and Web of Science. The search strategy integrated controlled vocabulary and free-text keywords with Boolean operators, focusing on four conceptual clusters: (1) UC-related terms (“ulcerative colitis” OR “UC”), (2) pyroptosis (“pyroptosis” OR “gasdermin D” OR “caspase-1”), (3) NLRP3 (“NLRP3” OR “NOD-like receptor protein 3”), and (4) therapeutic interventions (“natural products” OR “traditional Chinese medicine” OR “TCM” OR “herb”). The search was restricted to English-language original research and review articles, while excluding non-peer-reviewed publications and conference proceedings during preliminary screening.

## 3 Pyroptosis and its mechanisms

Pyroptosis is a form of programmed cell death (Coll et al., 2022). This process is fundamentally different from other types of cell death, such as apoptosis and necrosis, owing to its inflammatory consequences and its role in host defense mechanisms (Ketelut-Carneiro and Fitzgerald, 2022). Traditionally recognized as a response to infection, pyroptosis has been implicated in a range of pathologies, including inflammatory disorders (Dai Z. et al., 2023), cancer (Yang et al., 2022), and neurodegenerative diseases (Voet et al., 2019). Thus, a comprehensive understanding of the molecular mechanisms governing pyroptosis could provide new opportunities to find natural products that target pyroptosis for the treatment of UC.

### 3.1 Activation of inflammasomes

The onset of pyroptosis is mainly regulated by the activation of inflammasomes, which are multiprotein complexes (Man and Kanneganti, 2015). These inflammasomes are formed in response to various stimuli, including pathogen-associated molecular patterns (PAMPs) and damage-associated molecular patterns (DAMPs) (Zhan et al., 2022a). The most well-studied inflammasomes include NLRP3, absent in melanoma 2 (AIM2), and NLRC4, each responding to distinct signals. Upon detection of these signals, pattern recognition receptors (PRRs) such as NOD-like receptors (NLRs) and AIM2 oligomerize and recruit apoptosis-associated speck-like protein containing a CARD (ASC), forming a large protein scaffold. This recruitment facilitates the activation of caspase-1, an essential protease in the pyroptotic pathway. Activated caspase-1 cleaves pro-inflammatory cytokines such as pro-IL-1β and pro-IL-18 into their active forms, leading to their secretion and subsequent inflammatory response. Additionally, caspase-1 cleaves GSDMD, leading to the formation of pores in the cell membrane that characterize pyroptosis (Zhang W. J. et al., 2021; Zhan et al., 2022a).

### 3.2 Gasdermin family and pore formation

The gasdermin family of proteins plays a pivotal role in the execution of pyroptosis. Among them, GSDMD has emerged as the central player. Upon cleavage by caspase-1, the N-terminal domain of GSDMD translocates to the plasma membrane, where it oligomerizes and forms large pores. These pores facilitate the influx of water and ions, causing cell swelling and eventual lysis (Shi et al., 2017; Broz et al., 2020). This contrasts with apoptosis, where cell shrinkage and membrane blebbing occur without lysis. Recent studies have also identified other gasdermin family members, such as GSDME (gasdermin E), which can mediate pyroptosis in a caspase-3 dependent manner, linking apoptosis and pyroptosis in certain contexts. Gasdermins serve a dual function in apoptosis and pyroptosis, showcasing a complex interaction that can affect cell fate, which is dependent on the surrounding microenvironment and the nature of the cellular stress encountered (Jiang et al., 2020).

### 3.3 Role of cytokines in pyroptosis

The release of pro-inflammatory cytokines during pyroptosis not only contributes to local inflammation but also influences systemic responses. IL-1β and IL-18 are the hallmark cytokines released during pyroptosis, and their secretion can amplify inflammatory responses, recruit immune cells, and promote further pyroptotic cell death in neighboring cells (Zheng et al., 2023). Cytokines play a key role in the inflammatory response, but their activation and release are tightly controlled to maintain organismal homeostasis. Disruption of this regulation may play a role in the emergence of several pathological conditions, such as autoinflammatory syndromes and chronic inflammatory diseases (Tan et al., 2021; Atabaki et al., 2023).

**FIGURE 1:**
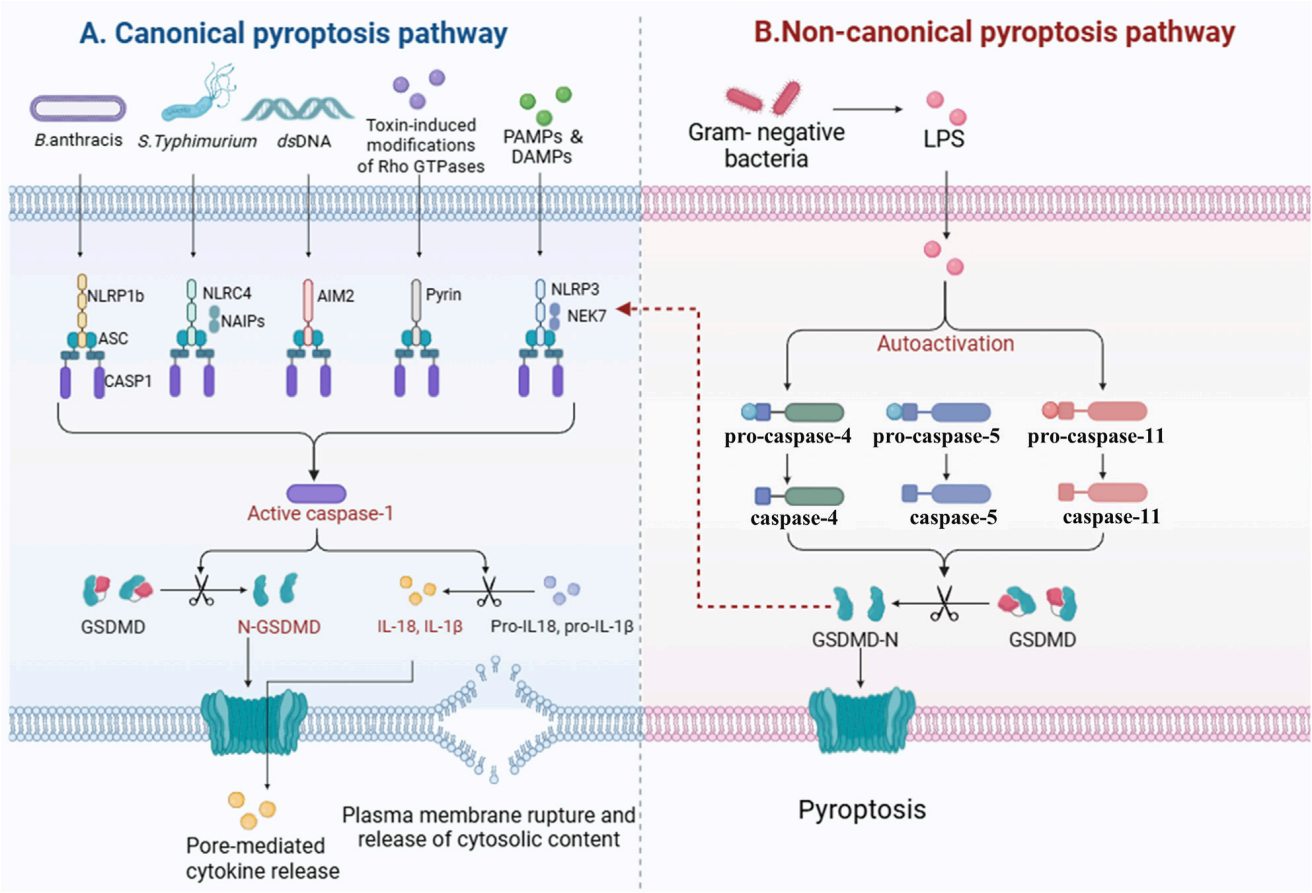
Mechanisms of pyroptosis activation. This diagram illustrates the two primary pathways involved in the initiation of pyroptosis: the canonical and non-canonical pathways. The canonical pyroptosis pathway is activated by the detection of pathogen-associated molecular patterns (PAMPs) or damage-associated molecular patterns (DAMPs), leading to the assembly of inflammasomes and the activation of caspase-1 **(A)**. In contrast, the non-canonical pyroptosis pathway is triggered by the intracellular sensing of bacterial lipopolysaccharides (LPS), resulting in the activation of caspase-4, -5, or -11 **(B)**. Both pathways converge to elicit a robust pro-inflammatory response characterized by the maturation and secretion of cytokines such as IL-1β and IL-18. This inflammatory response ultimately leads to the permeabilization of the cell membrane and the execution of cell death through pyroptosis.

In essence, the process of pyroptosis is typically initiated through the assembly of inflammasomes, which are critical components of the innate immune system. The activation of these inflammasomes may take place through two major routes: the canonical pathway (Figure 1A) and the non-canonical pathway (Figure 1B). The canonical route is chiefly marked by the identification of PAMPs or DAMPs, which leads to the activation of caspase-1. Conversely, the non-canonical route can be initiated by the intracellular detection of specific bacterial lipopolysaccharides, which activate caspase-4, -5, or -11. Ultimately, both pathways converge, resulting in a pro-inflammatory response that stimulates the maturation and secretion of cytokines like IL-1β and IL-18, alongside the disruption of the cell membrane, culminating in cell death. This mechanism not only acts as a defense mechanism against infections but also contributes to the modulation of the inflammatory environment in various disease conditions.

## 4 Pyroptosis in UC

The pathogenesis of UC is multifactorial and includes genetic susceptibility, environmental triggers, and dysregulated immune responses (Digby-Bell et al., 2020; Peng J. et al., 2024). Increasing evidence suggests that pyroptosis plays an important role in the inflammatory process associated with UC (Yang et al., 2024).

### 4.1 The role of pyroptosis in the pathogenesis and inflammation of UC

**FIGURE 2:**
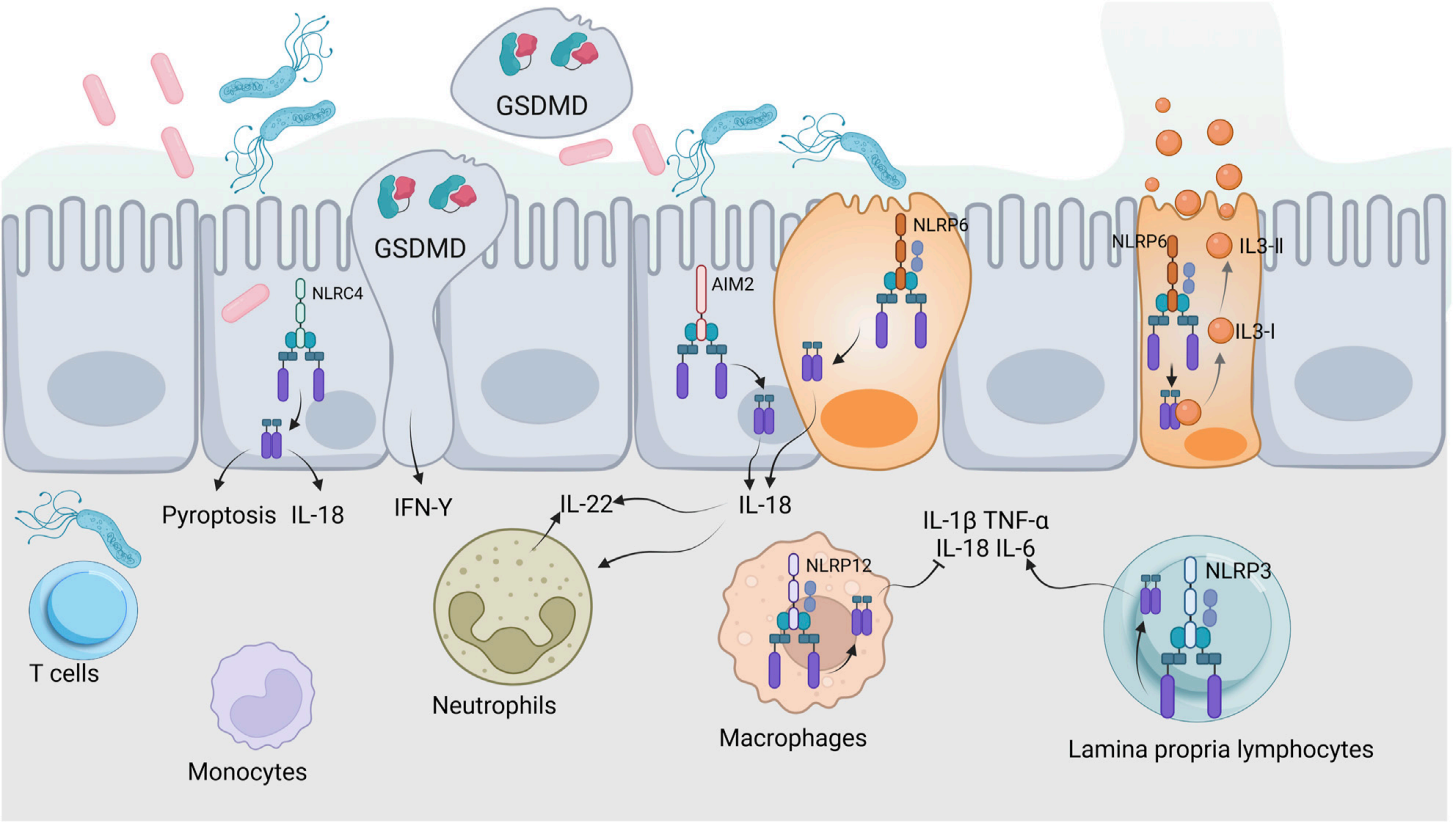
Inflammasomes and intestinal mucosal immunity. This illustration depicts the activation of inflammasomes (NLRP6, NLRC4, AIM2, NLRP12, NLRP3) in response to pathogens. Key processes include IL-18 secretion, IL-22 production, pyroptosis, and mucin secretion, highlighting their roles in antimicrobial defense and maintaining intestinal epithelial integrity.

Pyroptosis plays a critical role in the pathogenesis and inflammation of UC, as evidenced by elevated levels of caspase-1 and IL-1β in the colonic tissues of UC patients, indicating active pyroptotic processes (Gong et al., 2018). The activation of the NLRP3 inflammasome plays a crucial role in worsening colonic inflammation by triggering the release of pro-inflammatory cytokines and chemokines, which sustains the inflammatory process (Bian et al., 2024a). Additionally, dysregulation of pyroptosis has been linked to impaired epithelial barrier function in UC, allowing for increased microbial translocation and further immune activation (Yang et al., 2024). This relationship extends to the gut microbiome, which is crucial for maintaining intestinal homeostasis; dysbiosis is commonly associated with UC. Pyroptosis can change the composition and function of the gut microbiota by releasing inflammatory cytokines and damage-associated signals, which may lead to a cycle of inflammation and dysbiosis. On the other hand, certain microbial metabolites can influence inflammasome activation and pyroptosis, demonstrating a bidirectional relationship (Pan H. et al., 2022; Zhang Y. et al., 2024). Upon pathogen invasion, inflammasomes such as NLRP6, NLRC4, and AIM2 in epithelial cells are activated, leading to the secretion of IL-18. This cytokine stimulates intestinal epithelial or immune cells to produce IL-22, which promotes the production of antimicrobial peptides and proteins necessary for intestinal epithelial repair. The activation of inflammasomes can also induce pyroptosis through the assembly of GSDMD protein, forming membrane pores that cause cell swelling and death, thereby releasing intracellular bacteria and stimulating an immune response to enhance mucosal defense. The NLRP6 inflammasome in goblet cells not only facilitates IL-18 secretion and pyroptosis but also regulates autophagy to promote mucin protein secretion, helping to maintain intestinal epithelial integrity. Meanwhile, the NLRP12 inflammasome primarily modulates immune signaling pathways to prevent excessive inflammation. The NLRP3 inflammasome plays a dual role in lamina propria immune cells, activating inflammatory responses to bolster mucosal defense under homeostatic conditions, yet contributing to pathological damage during excessive infections (Figure 2) (Rathinam et al., 2019; Liu M. et al., 2023; Liu X. et al., 2023). The complex relationship among pyroptosis, immune regulation and inflammation highlights the potential for targeting these pathways in the development of therapeutic strategies for UC.

### 4.2 Potential biomarkers for pyroptosis in UC

Potential biomarkers discovery is essential for prognosis, diagnosis, and monitoring of disease activity in UC (Wangchuk et al., 2024). Recent studies have identified several potential biomarkers linked to pyroptosis that may be useful in clinical settings. Elevated levels of active caspase-1 in colonic tissue have been associated with increased inflammation in UC (Zhou et al., 2023b). Detection of caspase-1 activity provides insight into the extent of pyroptosis and inflammation and helps to grade patients according to the severity of the disease. Pro-inflammatory cytokines such as IL-1β and IL-18 are released during pyrolysis and are frequently elevated in patients with UC. Monitoring serum levels of these cytokines could serve as a reliable biomarker for assessing disease activity and response to therapy (Wang et al., 2020). For instance, a significant decrease in IL-1β levels following treatment might indicate effective modulation of the inflammatory process (Dai Y. et al., 2023; Ren et al., 2024). GSDMD as a key executor of pyroptosis, the presence of cleaved GSDMD in stool samples or plasma could serve as a novel biomarker for pyroptosis in UC. Its measurement could help determine the degree of pyroptotic activity in the intestinal epithelium, providing a direct link to epithelial cell death and inflammation (Lin et al., 2024a). The gut microbiome plays a pivotal role in regulating inflammation through the production of various metabolites. Certain microbial metabolites, such as short-chain fatty acids (SCFAs), may influence pyroptosis and could be explored as biomarkers reflecting the state of the microbiome and its interaction with the host immune response (Lv et al., 2024). Assessing the infiltration of specific immune cell types, such as neutrophils and Th17 cells, in colonic biopsies may also provide indirect biomarkers of pyroptotic activity. The presence of these cells correlates with inflammation and could indicate ongoing pyroptotic processes (Stakenborg et al., 2020).

### 4.3 Therapeutic targets within the pyroptotic pathway

Targeting the pyroptotic pathway offers a promising strategy for developing new therapeutic interventions for UC. Several potential therapeutic targets have emerged, including caspase-1 inhibitors, the preclinical studies have shown that inhibiting caspase-1 can reduce the severity of colitis in animal models, suggesting that pharmacological modulation of this enzyme may provide therapeutic benefits (Cao et al., 2024). Additionally, targeting the NLRP3 inflammasome directly presents another avenue for intervention. Compounds that inhibit NLRP3 activation could potentially reduce the inflammatory response in UC. For instance, small molecules or natural compounds that disrupt the assembly of the inflammasome complex might prevent downstream cascades leading to pyroptosis (Chen et al., 2021). Moreover, since GSDMD is a crucial executor of pyroptosis, therapies aimed at inhibiting its activity may help control excessive inflammation. Understanding the structural dynamics of GSDMD and its interaction with caspases could lead to the development of targeted inhibitors that prevent pore formation and subsequent cell lysis (Wang et al., 2021). Given the central role of IL-1β and IL-18 in promoting inflammation, targeted therapies that block these cytokines could be beneficial. Existing IL-1β antagonists (e.g., anakinra) and IL-18 binding proteins are being explored in various inflammatory conditions and could be repurposed for UC treatment (Peiró et al., 2017; Liso et al., 2022; Park et al., 2022). Furthermore, restoring a healthy gut microbiome through probiotics, prebiotics, and dietary interventions may influence pyroptosis indirectly by balancing immune responses (Darb Emamie et al., 2021; LeBlanc et al., 2021). In short, combination therapies targeting multiple components of the pyroptotic pathway, alongside traditional immunosuppressive agents, may offer a more effective treatment approach than single-agent therapies.

## 5 Mechanisms of pyroptosis regulation by natural products in UC

In recent years, natural products derived from plants have attracted much attention because of their potential to target pyroptosis in the treatment of UC (Xue et al., 2023; Chen B. et al., 2024). Among them, alkaloids, such as berberine, also contribute to UC management through their gut-protective and anti-inflammatory actions (Zhao X. et al., 2022). Polyphenols, abundant in fruits, vegetables, and so on, demonstrate significant anti-inflammatory effects, with notable examples being resveratrol (Wang S. et al., 2022). Additionally, flavonoids have been extensively studied for their notable anti-inflammatory and antioxidant properties (Fan et al., 2022). The mechanisms by which these natural products act include inhibition of inflammasome activation, particularly the NLRP3 inflammasome, thereby reducing the release of proinflammatory cytokines such as IL-1β and IL-18. In addition, they can regulate the release of inflammatory cytokines by down-regulating the NF-κB signaling pathway, thereby promoting a more balanced immune response. Additionally, many natural products enhance epithelial cell survival and intestinal barrier function, protecting against oxidative stress and inflammation, which helps maintain gut integrity and prevent exacerbation of inflammatory responses. Collectively, these findings underscore the promising role of natural products in the therapeutic management of UC through the modulation of pyroptosis. Therefore, this part summaries the role and regulatory mechanisms of several classes of natural products, including flavonoids, polyphenols and alkaloids, among others, in the treatment of UC through influencing pyroptosis. The detailed dosages and mechanisms of these natural products are summarized in Table 1 and their mechanisms are summarized in Figure 4A.

**TABLE 1:** Targeting pyroptosis for the treatment of UC with natural products.

Types	Compounds	Dosages	Cellular/animal models	Mechanisms	Ref.
Flavonoids	Quercetin	10, 20, 50 μM	LPS/ATP-induced THP-1 macrophage pyroptosis	Via TLR2/Myd88/NF-κB and ROS/AMPK pathway	Luo et al. (2022)
	Quercetin	100 mg/kg	2.5% DSS-induced UC mice	By reducing the expression level of TLR4, inhibiting cell pyroptosis, and improving intestinal inflammatory response	Zhao et al. (2024d)
	Hydroxysafflor yellow A	*In vivo*: 30, 60 mg/kg; *In vitro*: 40, 80,160 μM	3% DSS-induced UC mice; 1 μg/mL LPS-induced THP-1 cells	By suppressing pyroptosis *via* inhibiting HK1/NLRP3/GSDMD and modulating gut microbiota	Chen et al. (2023b)
	Jaceosidin	5, 10, 20, 40 μM	5 μg/mL LPS-induced NCM460 cells	By regulating NLRP3-mediated pyroptosis through activating SIRT1/NRF2 and ameliorating intestinal epithelial cell injury	Lv et al. (2023)
	Lonicerin	*In vivo*: 3, 10, 30 mg/kg; *In vitro*: 1, 3, 10, 30 μmol/L	2.5% DSS-induced UC mice; BMDMs and PMA-differentiated THP-1 cells	By autophagy-mediated NLRP3 inflammasome inactivation	Lv et al. (2021)
	Phloretin	25, 50, 100 mg/kg	2.5% DSS-induced UC mice	By modulating NF-κB, TLR4, and PPARγ pathways and inhibit the activation of NLRP3 inflammasome	Zhang et al. (2019)
	Phloretin	60 mg/kg	3% DSS-induced UC mice	By inhibiting NF-κB and NLRP3 inflammasome activation, ameliorating the oxidant stress and regulating the gut microbiota	Wu et al. (2019)
	Pelargonidin-3-galactoside	25 mg/kg	3% DSS-induced UC mice	By suppressing pyroptosis and altering intestinal flora structure	Chen et al. (2024b)
	Naringin	*In vivo*: 25, 50, 100 mg/kg; *In vitro*: 20 μM	2.5% DSS-induced UC mice; LPS-induced RAW264.7 cells	By activating PPARγ, suppressing NLRP3 inflammasome activation, and regulating ZO-1 expression	Cao et al. (2018)
	Oroxindin	*In vivo*: 12.5, 25, or 50 mg/kg; *In vitro*: 12.5, 25, 50 μM	3% DSS-induced UC mice; LPS-induced THP-1 cells	Via suppressing TXNIP-dependent NF-κB pathway	Liu et al. (2020b)
	Rutin	12.5, 25, 50 μmol/L	LPS-stimulated FHCs cells	By inhibiting NLRP3 inflammasome signaling pathway	Zhao et al. (2024c)
	Trifolirhizin	12.5, 25, 50 mg/kg	1.5% DSS-induced UC mice	Through inhibiting the TXNIP-mediated activation of NLRP3 inflammasome	Zhang et al. (2022b)
	Carthamin yellow	20, 40 mg/kg	2.5% DSS-induced UC mice	By repairing the intestinal barrier and activating the Nrf2/GPX4 axis	Bian et al. (2024b)
	Galangin	*In vitro*: 2.5, 5, 10 μM; *In vivo*: 40 mg/kg	HEK293 cells; LPS/ATP-induced HT29 cells; 2.5% DSS-induced UC mice	By targeting HSP90β, which regulates fatty acid synthesis and subsequently modulates NLRP3 inflammasome activation	Yang et al. (2023)
	Nicotiflorin	*In vivo*: 100, 300 mg/kg; *In vitro*: 50, 100 μM	LPS/ATP-induced RAW264.7 cells; LPS/ATP-induced BMDMs cells; 3% DSS-induced UC mice	By regulating activation of NLRP3 inflammasome and inhibit the inflammatory response by targeting p65 and inhibiting the NF-κB pathway	Ruan et al. (2024)
Phenylpropanoids	Honokiol	*In vivo*: 2.5, 5 mg/kg; *In vitro*: 5, 10 μM	3% DSS-induced UC mice; 100 ng/mL LPS-induced RAW264.7 macrophages	By targeting PPAR-γ-TLR4-NF-κB signaling and suppressing gasdermin-D-mediated pyroptosis	Wang et al. (2022b)
	Isofraxidin	*In vivo*: 10, 20, 40, 80, 160 μM; *In vitro*: 20, 40, 80 mg/kg	LPS-induced inflammation in HIEC cells; 4 g/100 mL DSS-induced UC mice	Through upregulating Nrf2, promoting its entry into the nucleus, and reducing ROS	He et al. (2024)
	Schisandrin B	*In vivo*: 10 mg/kg; *In vitro*: 40 μM	2% DSS-induced UC mice; LPS/ATP-stimulated HCT-116 cells	Through regulating pyroptosis by AMPK/Nrf2/NLRP3 inflammasome	Zhang et al. (2021b)
	Chlorogenic acid	*In vivo*: 20, 40 mg/kg; *In vitro*: 62.5 μM	3% DSS-induced UC mice; LPS/ATP-induced RAW264.7 cells	By downregulating miR-155 expression and inactivating the NF-κB/NLRP3 inflammasome pathway	Zeng et al. (2020)
	Schisandrin	20, 40, 80 mg/kg	3% DSS-induced UC mice	By inhibiting the SGK1/NLRP3 signaling pathway and reshaping gut microbiota	Wang et al. (2023c)
	Nodakenin	10, 20, 40 mg/kg	2.5% TNBS containing 50% ethanol-induced UC mice	By suppressing NFƙB-mediated NLRP3 inflammasome pathway	Shah and Solanki (2024b)
	Fraxetin	10, 30, 60 mg/kg	2% DSS-induced UC mice	By modulating the inflammatory response, enhancing epithelial barrier integrity and regulating the gut microbiota	Sun et al. (2024b)
	Cinnamaldehyde	*In vivo*: 20, 40, 80 mg/kg; *In vitro*: 20, 40, 80 μM	3% DSS-induced UC mice; LPS-induced RAW264.7 cells	by modulating TLR4/NF-кB signaling pathway and NLRP3 inflammasome activation	Tan et al. (2023)
	Ferulic acid	*In vivo*: 10, 20, 250 mg/kg; *In vitro*: 125, 250, 500 μM	100 mg/kg TNBS-induced UC rat; TNF-α-induced HIMECs	By inhibiting the TXNIP/NLRP3 pathway	Yu et al. (2023b)
	Sinapic acid	40, 80 mg/kg	2.5% DSS-induced UC mice	By modulating NLRP3 inflammasome activation and the autophagy pathway	Li et al. (2024d)
Polyphenols	Resveratrol	25 and 50 μM	LPS/ATP-induced HT29 cells	NF-κB pathway	Zhao et al. (2024b)
	Gallic acid	*In vivo*: 40, 80, 120 mg/kg; *In vitro*: 50, 100, 200 μM	3.5% DSS-induced UC mice; LPS-stimulated RAW264.7 cells	Via inhibiting NLRP3 inflammasome	Yu et al. (2023c)
	Atranorin	*In vitro*: 25, 50, 100 μM; *In vivo*: 10 mg/kg	LPS/ATP/Nigericin/MSU-stimulated BMDM/BMDC/RAW264.7 cells; 5 mg/kg LPS-induced acute inflammation model; 200 μg MSU crystal induced gouty arthritis model; 3% DSS-induced UC mice	By suppressing NLRP3 inflammation activation through binding to ASC and inhibiting ASC oligomerization without influencing the priming stage	Wang et al. (2023a)
	F. suspensa polyphenols	*In vitro*: 62.5, 125.0, 250.0 μg/mL; *In vivo*: 200, 400, 600 mg/kg	5 μg/mL LPS and 20 ng/mL IFN-γ-stimulated mouse monocyte macrophage J774A.1; 2.5% DSS-induced UC mice	By regulating macrophage polarization from M1 to M2, which related to pyroptosis and gut microbiota	Lv et al. (2024)
	Apple Polyphenols Extract	125, 500 mg/kg	3% DSS-induced UC mice	Via inhibition of apoptosis and pyroptosis	Liu et al. (2021)
	Thyme (Thymus vulgaris L.) polyphenols	100, 200, 400 mg/kg	2% DSS-induced UC mice	By mitigating intestinal barrier damage, regulating gut microbiota, and suppressing TLR4/NF-κB-NLRP3 inflammasome pathways	Zhou et al. (2023a)
	Polyphenol Extracts from *Ziziphus jujuba* Mill. (PER)	0.25 mL of 200 mg/mL PER	3% DSS-induced UC mice	By inhibiting the NLRP3 and MAPKs signaling pathways and regulating gut microbiota homeostasis	Wei et al. (2024)
Alkaloids	Oxymatrine	*In vivo*: 40, 80 mg/kg; *In* *vitro*: 50, 100, 250 μM	The UC rat models were established by site-specific colonic injection of 35 mg/kg TNBS in 40% ethanol 8 cm away from the anus through a silicone tube; LPS/ATP-induced primary peritoneal macrophages and RAW326.7 cells pyroptosis	Inhibit pyroptosis mediated by the NLRP3 inflammasome	Sun et al. (2024a)
	Nigeglanine	*In vivo*: 10, 50, 100 mg/kg; *In vitro*: 1, 5, 10 μg/mL	3% DSS-induced UC mice; DSS-stimulated Caco-2 cells	By regulating inflammatory pathways (NF-κB and MAPKs pathways), inhibiting pyroptosis, and preserving intestinal barrier integrity	Gao et al. (2019)
	Berberine	*In vivo*: 20 mg/kg; *In vitro*: 50 μM	3% DSS-induced UC mice; 10 ng/mL IL-6-stimulated Caco-2 cells and NCM460 cells	By depressing Wnt/beta-catenin pathway activation *via* controlling the miR-103a-3p/BRD4 axis	Zhao et al. (2022a)
	Evodiamine	20, 40, 80 mg/kg	2.5% DSS-induced UC mice	Via the regulation of NF-κB and NLRP3 inflammasome	Shen et al. (2019)
	Nigakinone	*In vivo*: 25, 50, 60, 100 mg/kg; *In vitro*: 0.2, 1, 5 μM	4% DSS-induced UC mice; CT26 cells	Via regulating bile acid profile and FXR/NLRP3 signaling pathways	Liu et al. (2023b)
	Gentianine	12.5, 25, 50 mg/kg	3% DSS-induced UC mice; 500 ng/mL LPS-induced HT-29 cells	By inhibiting TLR4/NLRP3-mediated pyroptosis	Li et al. (2024e)
	8-Oxypalmatine	12.5, 25, 50 mg/kg	3% DSS-induced UC mice	Via regulating Nrf2 and NLRP3 inflammasome	Cheng et al. (2022)
	Aegeline	5, 10, 20 mg/kg	100 μL TNBS containing 50% ethanol-induced UC mice	By suppressing the NF-κB-mediated NLRP3 inflammasome pathway	Shah and Solanki (2024a)
	Oxymatrine	50, 100 mg/kg	4% DSS-induced UC mice	By improving ferroptosis and inflammation, mainly target to the expression of IL-1β, IL-6, NOS2, HIF1A, IDO1, HMGB1, and NLRP3	Gao et al. (2024)
	Sanguinarine	*In vivo*: 5, 10 mg/kg; *In vitro*: 0.25, 0.5, 1.0 μM	3% DSS-induced UC mice; LPS-induced THP-1 cells	By inhibiting NLRP3 inflammasome activation and modulating intestinal microbiota	Li et al. (2022d)
	Coptisine	50, 100 mg/kg	3% DSS-induced UC mice	Through modulating gut microbiota and inhibiting TXNIP/NLRP3 inflammasome	Li et al. (2024a)
	Demethyleneberberine	*In vivo*: 100, 200 mg/kg; *In vitro*: 10 μM	3% DSS-induced UC mice; LPS/ATP-induced RAW264.7 cells; Primary intestinal macrophages from fetal mouse; HcoEpiC cells	By blocking the maturation of IL-1β in inflammation through inhibiting TLR4-mitochondria signaling	Zhao et al. (2022b)
	Betaine	600 mg/kg	4% DSS-induced UC mice	By inhibiting oxidative stress induced inflammatory pyroptosis	Chen et al. (2022)
Terpenoids	Brusatol	*In vitro*: 25, 50, 100 nM; *In* *vivo*: 0.25, 0.5, 1.0 mg/kg	LPS-induced RAW326.7 cells; 25 mg/kg TNBS containing 50% ethanol-induced UC mice	Via suppression of NF-κB and NLRP3-mediated inflammatory responses, and regulation of Nrf2-mediated oxidative stress	Zhou et al. (2018)
	Ginsenoside Rg3	*In vitro*: 5, 10 mg/mL; *In vivo*: 10 mg/kg	1 μg/mL LPS-induced CECs/BMDMs cells; 3% DSS-induced UC mice	By inhibiting NLRP3 inflammasome activation and restoring gut microbiota homeostasis	Liu et al. (2023a)
	Artemisinin analog SM934	*In vivo*: 10 mg/kg; *In vitro*: 10 μM	2.5% TNBS containing 50% ethanol-induced UC mice; TNF-α-stimulated Caco-2 cells; LPS/ATP-stimulated HT-29 cells	Via inhibiting apoptosis and caspase-1-mediated pyroptosis	Shao et al. (2022)
	Celastrol	1 mg/kg	4% DSS-induced UC mice	CSR inhibits the priming step of the NLRP3 inflammasome by disrupting NF-κB signaling and downregulating HSP-90 at both protein and mRNA levels, while CP further suppresses NLRP3 expression	Saber et al. (2020)
	20(S)- Protopanaxadiol saponins	*In vivo*: 50, 100, 200 mg/kg; *In vitro*: 30, 60 μM	3% DSS-induced UC mice; PMA-activated THP-1 macrophages; LPS-induced HT29 cells; co-cultured model of HT29 and THP-1 macrophages	By inhibiting the binding of HMGB1 to TLR4 and following NF-κB/NLRP3 inflammasome pathway activation	Chen et al. (2023a)
	Bryodulcosigenin	*In vivo*: 10 mg/kg; *In vitro*: 10 μM	2.5% DSS-induced UC mice; TNF-α-induced NCM460 cells and MLE-12 cells (co-culture)	By preventing apoptosis in intestinal epithelial cells and inhibited the activation of the NLRP3 inflammasome, leading to the restoration of the intestinal barrier function	Li et al. (2022c)
	Mogrol	*In vitro*: 1, 10 μM; *In vivo*: 1, 5 mg/kg	Phorbol myristate acetate-stimulated THP-1 cells to differentiate into macrophages (THP-Ms), LPS/ATP-stimulated THP-M cells; TNF-α-induced NCM460 cells; 4% DSS-induced UC mice	by activating AMPK signaling pathway	Liang et al. (2021)
	1,8-Cineole	*In vivo*: 1, 5 mg/kg; *In vitro*: 50, 100, 200 μM	2.5% DSS-induced UC mice; LPS/IFNγ-induced RAW264.7 and BMDMs; Caco2/THP-1 coculture model	Via inhibiting the HSP90-NLRP3-SGT1 complex	Ma et al. (2023b)
	*Pulsatilla chinensis* saponins	300 mg/kg	4 g/kg DSS-induced UC mice	By increase the content of colonic SCFAs, activating the GPR43-NLRP3 signaling pathway, and reducing the levels of pro-inflammatory cytokines	Li et al. (2023)
Steroids	β-sitosterol	*In vivo*: 50, 100, 200 mg/kg; *In vitro*: 8, 16 μg/mL	4% DSS-induced UC mice; 50 mg/mL DSS-stimulated Caco-2 cells	NLRP3/Caspase-1/GSDMD-mediated pyroptosis and inflammation response	Zhang et al. (2023)
	Ruscogenin	*In vitro*: 2.5, 5, 10 μM; *In vivo*: 0.5, 1, 2 mg/kg	3.5% DSS-induced UC mice; LPS/Nigericin-induced THP-1 cells	By inhibiting caspase-1-dependent pyroptosis *via* the TLR4/NF-κB signaling pathway	Li et al. (2024c)
	Dioscin	*In vivo*: 40 mg/kg; *In vitro*: 600 ng/mL	2.5% DSS-induced UC mice; LPS-induced primary mouse peritoneal Macrophage	By regulating the polarization of intestinal M1/M2 macrophages and NF-κB, MAPK pathway and NLRP3 activation	Cai et al. (2021)
	Physalin B	*In vitro*: 25, 50, 100 μM; *In vivo*: 10 mg/kg	LPS-induced RAW 264.7 cells; 4% DSS-induced UC mice	By suppressing the activation of NF-κB, STAT3, β-arrestin1 and NLRP3 inflammasome	Zhang et al. (2020b)
Others	Diacetylrhein	50, 100 mg/kg	4% DSS-induced UC rat	By inhibiting the NLRP3 inflammasome, reducing caspase-1 activity and inflammatory cytokines, altering apoptotic protein balance, disrupting NFκB signaling, and enhancing tight junction protein expression	Zohny et al. (2022)
	Salidroside	*In vivo*: 7.5, 10, 15 mg/kg; *In vitro*: 10, 20, 40, 80 μM	2.5% DSS-induced UC mice; LPS/ATP-induced Caco-2/BMDMs cells	Via inhibiting macrophage pyroptosis and repairing the dysbacteriosis-associated Th17/Treg imbalance	Liu et al. (2023e)
	Salidroside	15 mg/kg	2.5% DSS-induced UC mice	By maintaining the intestinal barrier while effectively restoring the balance between inflammasome and autophagy	Liu et al. (2019)
	Pectic polysaccharides from Rauwolfia verticillata var. Hainanensis	100 mg/kg	5% DSS-induced UC mice	By suppressing the expression of caspase-1 and IL-1β	Cui et al. (2022)
	Pectic polysaccharides from Rauwolfia verticillata var. Hainanensis	*In vivo*: 100 mg/kg; *In* *vitro*: 640 μg/mL	4% DSS-induced UC mice; 640 μg/mL LPS-induced YAMC cells	By upregulating miR-124–3p expression and reduced the binding of RBP4 to NLRP3 to inhibit NLRP3-mediated colonic epithelial cell pyroptosis	Yuan et al. (2023)
	Polysaccharides (DOPS) from Dendrobium officinale	*In vivo*: 5, 100, 200 mg/kg; *In vitro*: 5, 100, 200 μg/mL	4% DSS-induced UC mice; LPS-stimulated NCM460 cells	By inhibiting the NLRP3 inflammasome and β-arrestin1 signaling pathways	Liang et al. (2018)
	Trans-10-Hydroxy-2-Decenoic Acid (10-HDA) in royal jelly	25, 100 mg/kg; 0.5, 1, 2 mM	2.5% DSS-induced UC mice; LPS/ATP-stimulated THP-1 cells	By regulating the NLRP3 inflammasome-mediated pyroptotic pathway and enhancing colonic barrier function	Huang et al. (2022)
	3,5-Dimethyl-8-methoxy-3,4-dihydro-1 H-isochromen-6-ol	2.5, 5 mg/kg	3% DSS-induced UC mice	Via Targeting NLRP3	Zhou et al. (2024c)

### 5.1 Flavonoids

**FIGURE 3:**
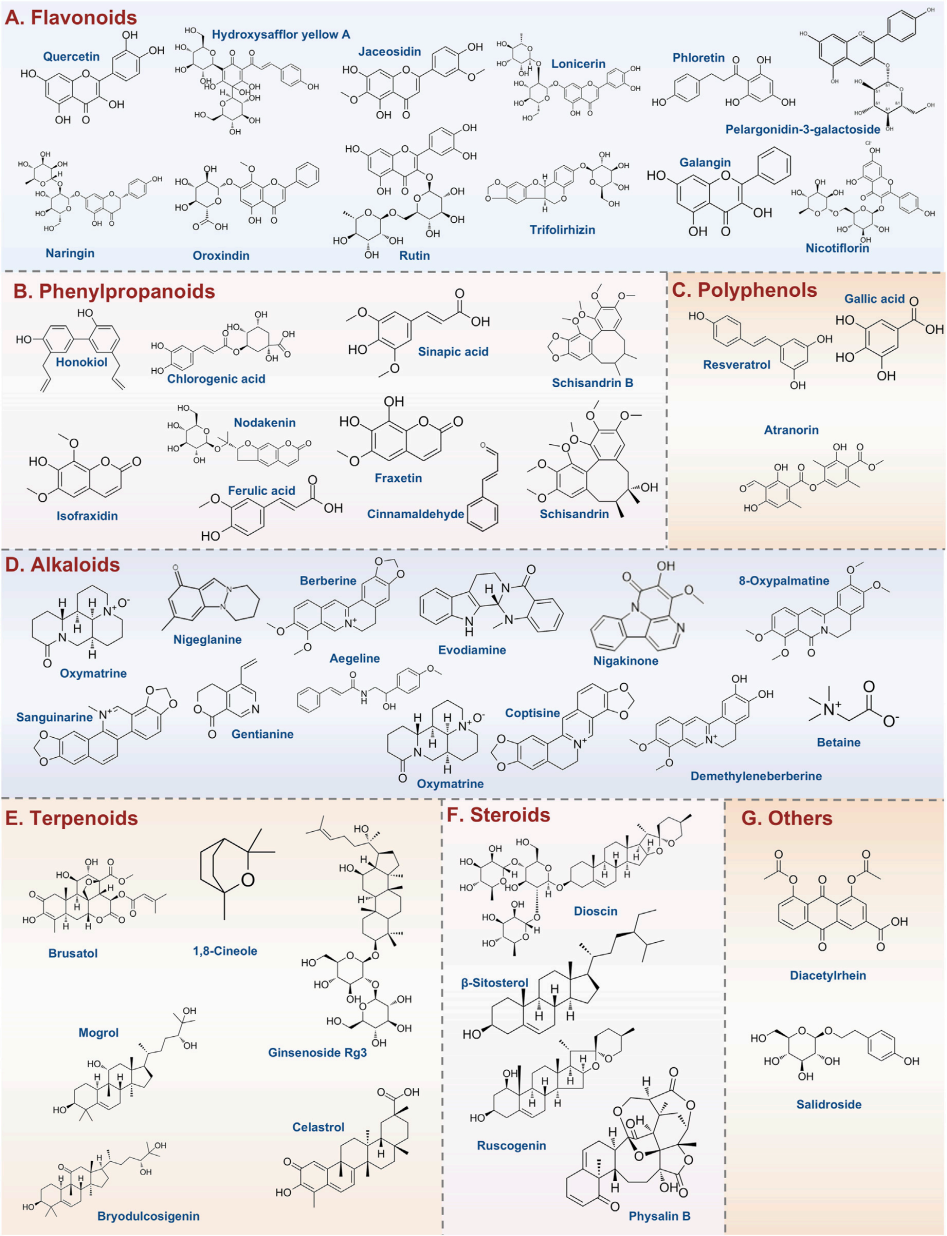
Types of natural products and representative compounds targeting pyroptosis for UC treatment. **(A)** Flavonoids; **(B)** Phenylpropanoids; **(C)** Polyphenols; **(D)** Alkaloids; **(E)** Terpenoids; **(F)** Steroids; **(G)** Others.

Flavonoids are a diverse group of polyphenolic compounds with a wide range of biological activities. Figure 3A illustrates the currently reported flavonoids that target pyroptosis for the treatment of UC. Among them, Quercetin is particularly notable for its multifaceted beneficial properties, which include antioxidant, antiviral, anticancer, anti-inflammatory, and antimicrobial effects. Notably, Quercetin has been shown to protect macrophages from pyroptosis, partially by inhibiting inflammasome activity (Luo et al., 2022). Furthermore, in models of UC, Quercetin has been reported to reduce the expression of TLR4, inhibit cellular pyroptosis, and ameliorate the intestinal inflammatory response, particularly during the active phase of the disease (Zhao Y. et al., 2024). Another significant flavonoid is Hydroxysafflor yellow A (HSYA), a primary water-soluble chalcone glycoside derived from *Carthamus tinctorius* L. Previous studies have demonstrated HSYA’s exceptional anti-inflammatory and antioxidant properties (Luo et al., 2020). It has been shown to decrease pro-inflammatory cytokines, such as IL-1β, IL-6, and TNF-α, while also inhibiting NLRP3/GSDMD-mediated pyroptosis *in vitro*. Additionally, HSYA downregulates hexokinase 1 (HK1), and *in vivo* studies indicate that it alleviates UC symptoms, including body weight loss and inflammatory infiltration, while modulating gut microbiota by reducing Proteobacteria and increasing Bacteroidetes (Chen et al., 2023b). Jaceosidin, a flavonoid extracted from *Artemisia princeps*, is recognized for its antioxidant, anti-inflammatory, and anticancer properties. It has been found to significantly inhibit LPS-induced oxidative stress and the accumulation of inflammatory cytokines, as well as NLRP3-mediated cell lysis in LPS-induced NCM460 cells. Moreover, jaceosidin activates the SIRT1/NRF2 pathway, leading to the inhibition of NLRP3-mediated pyroptosis, thereby protecting NCM460 cells from this form of cell death (Lv et al., 2023). Lonicerin, a flavonoid glycoside derived from *Lonicera japonica* Thunb, has been identified as a major component with anti-inflammatory and immunomodulatory activities (Lin et al., 2024b). Research indicates that lonicerin targets EZH2 to alleviate UC symptoms through the autophagy-mediated inactivation of NLRP3 inflammatory vesicles (Lv et al., 2021). Phloretin, a dihydrochalcone flavonoid primarily found in apples and strawberries, effectively suppresses pro-inflammatory cytokines by modulating the NF-κB, TLR4, and PPARγ signaling pathways. It also inhibits NLRP3 inflammasome activation and reduces markers of oxidative stress. Phloretin is shown to regulate the levels of zonula occludens-1 (ZO-1) and occludin, lower serum LPS concentrations, and rebalance the gut microbiota by modulating the populations of *Escherichia coli* and *Lactobacillus* (Zhang et al., 2019). Pelargonidin-3-galactoside (Pg3gal), derived from purple sweet potatoes, has been found to significantly alleviate DSS-induced UC in mice. Its protective effects include reducing inflammation, inhibiting pyroptosis in intestinal epithelial cells, and restoring the balance of gut microbiota (Chen J. et al., 2024). In addition to these key flavonoids, several other natural products have shown promise in the treatment of UC. Such as Naringin, Oroxindin, Rutin, Trifolirhizin, Carthamin yellow, Galangin, and Nicotiflorin. Collectively, these flavonoids demonstrate significant potential as therapeutic agents in the management of UC, highlighting their multifaceted roles in modulating inflammatory pathways and gut microbiota.

### 5.2 Phenylpropanoids

Figure 3B illustrates the currently reported phenylpropanoids that target pyroptosis for the treatment of UC. Honokiol, an active natural compound sourced from *Magnolia officinalis*, has a historical use in traditional medicine for addressing infections, inflammation, and gastrointestinal disorders (Lee et al., 2011). Recent studies indicate that honokiol significantly alleviates UC severity by reducing inflammatory responses and restoring colonic integrity. It has been shown to lower the levels of pro-inflammatory cytokines such as TNF-α, IL-6, IL-1β, and IFN-γ, while enhancing the expression of PPAR-γ. Furthermore, Honokiol inhibits the TLR4-NF-κB signaling pathway, a critical pathway in inflammation, and reduces gasdermin-D-mediated pyroptosis, indicating a robust anti-inflammatory profile that may offer a promising therapeutic strategy for UC management (Wang N. et al., 2022). Isofraxidin, possesses a diverse array of beneficial properties, including anti-inflammatory, antioxidant, anti-cancer, and anti-fatigue effects (Shen et al., 2017). Recent studies have demonstrated that Isofraxidin can alleviate DSS-induced UC by modulating the S1PR1 and IL-17 signaling pathways (Huang Y. et al., 2024). Furthermore, it has been shown to inhibit pyroptosis by upregulating the Nrf2 protein, facilitating its translocation into the nucleus, and reducing ROS levels (He et al., 2024). Schisandrin B, the primary active component of Schisandra chinensis, has exhibited a range of biological activities, including anticancer, anti-inflammatory, hepatoprotective, and antibacterial effects. Notably, Schisandrin B has been found to inhibit the activation of the NLRP3 inflammasome, leading to reduced levels of IL-1β and pyroptosis in intestinal epithelial cells within UC models. This protective effect is mediated through the activation of AMPK and Nrf2-dependent signaling pathways, as well as the alleviation of ROS-induced mitochondrial damage. These findings suggest that Schisandrin B represents a promising therapeutic strategy for the treatment of acute colitis (Zhang W. et al., 2021). In addition to the phenylpropanoids listed above, phenylpropanoids such as Schisandrin, Chlorogenic acid, Nodakenin, Fraxetin, Cinnamaldehyde, Ferulic acid and Sinapic acid, have shown promising potential for improving UC. Collectively, these phytochemicals collectively illuminate pyroptosis inhibition as a strategic axis in treating UC, while underscoring the therapeutic value of natural compounds in modulating inflammasome-immune crosstalk.

### 5.3 Polyphenols

Emerging evidence highlights the therapeutic potential of polyphenols in alleviating intestinal inflammation, particularly in the context of UC. Figure 3C illustrates the currently reported polyphenols that target pyroptosis for the treatment of UC. Resveratrol, a stilbene-polyphenol predominantly found in the skin of grapes and certain berries, possesses significant health benefits, including anti-inflammatory, neuroprotective, antioxidative, and anticancer properties (Zhang L. X. et al., 2021). Research has shown that Resveratrol can effectively inhibit LPS and ATP-induced sepsis in HT29 cells. This inhibition occurs through the downregulation of the NF-κB signaling pathway, leading to reduced expression of sepsis-associated proteins and inflammatory mediators. Such findings underscore the therapeutic potential of Resveratrol for managing UC (Zhao P. et al., 2024). Gallic acid (GA), a polyphenolic compound known for its broad pharmacological activities, particularly its anti-inflammatory effects, has shown promise in ameliorating DSS-induced UC by inhibiting the NLRP3 inflammasome (Yu T. Y. et al., 2023). Atranorin, a secondary metabolite derived from lichens, demonstrates significant anti-inflammatory properties by inhibiting nitric oxide production, leukotriene B4 biosynthesis, and cyclooxygenase-1 activity. Toxicity assays in rats have confirmed its safety for *in vivo* use. Atranorin has been identified as a novel inhibitor of the NLRP3 inflammasome, effectively blocking ASC oligomerization and cytokine release in macrophages and dendritic cells. In various disease models driven by inflammasome activation, such as acute inflammation, gouty arthritis, and UC, Atranorin has been shown to significantly reduce levels of IL-1β and IL-18, underscoring its therapeutic potential (Wang H. Y. et al., 2023). *Forsythia suspensa* polyphenols have demonstrated efficacy through a multifaceted approach, including the inhibition of M1 macrophage polarization, promotion of thermal apoptosis, and modulation of overall apoptosis. Additionally, these polyphenols facilitate M2 polarization in J774A.1 cells and exhibit protective effects against DSS-induced UC in murine models. They also play a critical role in regulating intestinal homeostasis by enhancing gut microbiota and increasing SCFAs production, key metabolites for gut health (Lv et al., 2024). Apple polyphenols extract (APE), sourced from red Fuji apples, has been attributed with multiple health-promoting effects, including anti-inflammatory, antioxidant, and anti-tumor properties. Recent studies have demonstrated that APE administration can ameliorate acute UC by preserving intestinal barrier integrity and inhibiting both apoptosis and pyroptosis (Liu et al., 2021). In addition to the polyphenols listed above, polyphenolic compounds such as Thyme (*Thymus vulgaris* L.) polyphenols and Polyphenol extracts from *Ziziphus jujuba* Mill. have shown promising potential for improving intestinal health and reducing intestinal inflammation. Collectively, these natural polyphenols provide new ideas for the treatment of UC through various mechanisms, such as modulation of immune response, improvement of microbiota, and antioxidant activity.

### 5.4 Alkaloids

Figure 3D illustrates the currently reported alkaloids that target pyroptosis for the treatment of UC. Oxymatrine, a principal alkaloid derived from *Sophora flavescens* Aiton of the Leguminosae family, exhibits a range of pharmacological effects, notably analgesic and anti-inflammatory properties (Chen et al., 2017; Sun J. et al., 2024). Recent studies have demonstrated that Oxymatrine effectively alleviates UC in TNBS-induced rat models by significantly reducing colonic ulcers and inhibiting pyroptosis via the NLRP3 inflammasome pathway. Specifically, treatment with Oxymatrine at a dosage of 80 mg/kg resulted in a notable decrease in levels of NLRP3, active caspase-1, and cleaved IL-1β in lesion tissues within a mere 24 h. *In vitro* experiments revealed that Oxymatrine at concentrations of 100 and 250 μM diminished cell death and lowered levels of active caspase-1, GSDMD, and cleaved IL-1β in RAW264.7 cells and peritoneal macrophages, underscoring its potential as a therapeutic strategy for UC (Sun J. et al., 2024). Another promising alkaloid, Nigeglanine, has been shown to inhibit pyroptosis in colonic epithelial cells by obstructing the NF-κB and MAPK signaling pathways, along with the NLRP3 inflammatory vesicles. Notably, treatment with Nigeglanine resulted in increased levels of ZO-1 and occludin proteins, which are crucial for maintaining intestinal barrier integrity, thereby potentially slowing the progression of UC (Gao et al., 2019). Berberine (BBR), recognized for its anti-inflammatory properties, is particularly relevant in the context of colitis management. Research indicates that Berberine can inhibit the activation of the Wnt/beta-catenin pathway through the modulation of the miR-103a-3p/BRD4 axis. This mechanism contributes to the attenuation of colitis-induced pyroptosis and aids in the restoration of intestinal mucosal barrier integrity (Zhao X. et al., 2022). Evodiamine (EVO), extracted from the traditional Chinese medicinal plant *Evodia rutaecarpa*, has been documented to possess a spectrum of pharmacological activities, including anti-inflammatory and anti-tumor effects (Li D. et al., 2022). EVO has been observed to significantly reduce the production of pro-inflammatory cytokines such as TNF-α, IL-1β, and IL-6, thereby suppressing inflammation associated with UC. This anti-inflammatory effect is mediated through the modulation of NF-κB signaling and the NLRP3 inflammatory vesicles (Shen et al., 2019). Additionally, Nigakinone, the primary active component of Ramulus et Folium Picrasmae, a traditional Chinese medicine (TCM) commonly used for treating dysentery, colitis, and gastroenteritis (Liu F. et al., 2020). Nigakinone exerts its protective effects by attenuating the inflammatory response linked to NLRP3 activation and by preserving the integrity of the intestinal mucosal barrier. Furthermore, it regulates bile acid metabolism and mitigates bile acid accumulation through FXR-mediated modulation of cholesterol hydroxylase. Molecular docking studies have suggested that FXR serves as a target for Nigakinone, and inhibition of FXR diminishes the protective effects of Nigakinone, highlighting its role in the modulation of the FXR/NLRP3 signaling pathway in the context of colitis (Liu F. et al., 2023). Gentianine (GTN), a prominent active compound derived from *Gentiana scabra*, has been recognized for its anti-inflammatory and anti-diabetic properties. Studies indicate that GTN effectively mitigates DSS-induced UC in mice and exhibits protective effects on inflamed colonic tissues *in vitro*. Notably, Toll-like receptor 4 (TLR4) has been identified as a direct target of GTN, suggesting that its therapeutic effects may stem from the modulation of TLR4, which subsequently reduces the activation of the NLRP3 inflammasome signaling pathway (Li Y. X. et al., 2024). Additionally, alkaloids such as 8-oxypalmatine, Aegeline, Sanguinarine, Coptisine, Brtaine, Betaine, and Demethyleneberberine have shown potential therapeutic effects in the context of UC and related inflammatory conditions. Collectively, this emerging evidence highlights that plant-derived alkaloids, including Oxymatrine, Nigeglanine, Berberine, and Evodiamine, exert therapeutic effects on UC by targeting pyroptosis through multi-pathway modulation of NLRP3 inflammasomes, NF-κB/MAPK signaling, and intestinal barrier restoration, offering novel strategies for inflammation resolution and mucosal repair.

### 5.5 Terpenoids

Figure 3E illustrates the currently reported terpenoids that target pyroptosis for the treatment of UC. The triterpene Celastrol (CSR) exhibits potent anti-inflammatory activities across multiple experimental models by disrupting NF-κB signaling pathways, which results in the downregulation of pro-inflammatory cytokines, including IL-1β. Combination therapy using CSR and the compoundCP-456773 has been shown to significantly improve colonic health by reducing colon shortening, disease activity index, and markers of inflammation. This combined treatment enhances antioxidant defenses, inhibits NLRP3 inflammasome activation through modulation of NF-κB and heat shock protein 90 (HSP-90), and promotes autophagy. These findings highlight the considerable potential of celastrol in treating inflammatory bowel disease and other conditions associated with NLRP3 inflammasome activation (Saber et al., 2020). In parallel, emerging studies highlight the therapeutic potential of Ginsenoside Rg3 in the management of DSS-induced UC. This compound alleviates colitis by inhibiting the activation of the NLRP3 inflammasome, as well as mitigating pyroptosis and apoptosis. Notably, Ginsenoside Rg3 also partially restores the disrupted gut microbiota at both the phylum and genus levels, indicating its modulatory impact on microbial composition. Additionally, Ginsenoside Rg3 appears to influence intestinal metabolism, suggesting a complex interplay between microbiota remodeling and inflammasome inhibition in its therapeutic effects against DSS-induced UC (Liu D. et al., 2023). Artemisinin, derived from *Artemisia annua*, exhibits potent anti-inflammatory and immunosuppressive effects against autoimmune diseases. Its novel water-soluble analog, SM934 (β-aminoarteether maleate), shows strong immunosuppressive activity with reduced side effects. In a study involving TNBS-induced colitis, SM934 effectively restored body weight and colon length while improving intestinal pathology. It protects intestinal barrier function by decreasing permeability, maintaining tight junction proteins and prevents epithelial cell apoptosis and pyroptosis. Furthermore, SM934 has been shown to inhibit the key cleavage-associated factors and block the MAPK and NF-kB signaling pathways in colonic tissues, providing a potential therapeutic avenue for treating UC (Shao et al., 2022). In addition, terpenoids components such as Brusatol, Bryodulcosigenin, 20(S)-Protopanaxadiol saponins, Mogrol, *Pulsatilla chinensis* saponins, and 1,8-cineole likewise exhibited significant anti-inflammatory and protective effects, which further enriches the potential of natural products in the treatment of UC diseases. In summary, these terpenoids demonstrate significant promise as therapeutic agents in the management of UC. Their various mechanisms, such as the modulation of important signaling pathways and the inhibition of pro-inflammatory cytokine production, provide a solid basis for further research and potential clinical use in treating inflammatory bowel diseases.

### 5.6 Steroids

Figure 3F illustrates the currently reported steroids that target pyroptosis for the treatment of UC. β-Sitosterol, a primary compound found in phytosterols, is a plant-derived natural product prevalent in various regions. Research indicates that β-sitosterol offers protective effects against (DSS-induced UC by mitigating weight loss and colon length reduction, alleviating macroscopic damage, decreasing pro-inflammatory factor production, and inhibiting NLRP3/caspase-1/GSDMD-mediated pyroptosis and inflammation. Furthermore, it enhances colonic barrier integrity, positioning it as a promising therapeutic agent for UC (Zhang et al., 2023). Ruscogenin, a significant steroidal glycoside extracted from *Radix Ophiopogon japonicus*, is recognized for its protective effects against inflammatory responses associated with various inflammatory diseases. Recent studies elucidate the potent protective effects of Ruscogenin against DSS-induced colitis, demonstrating its ability to alleviate the condition in mice by inhibiting the activation of the NLRP3 inflammasome and the caspase-1-dependent canonical pyroptosis. This protective mechanism is likely mediated through the inhibition of the TLR4/NF-κB signaling pathway (Li J. et al., 2024). Physalin B, a primary active withanolide extracted from *Physalis alkekengi* L. var. franchetii (Mast.) Makino, has demonstrated significant anti-inflammatory effects at non-cytotoxic doses. In LPS-stimulated cells, Physalin B notably reduced the levels of pro-inflammatory cytokines, including TNF-α, IL-6, and IL-1β. Furthermore, in a DSS-induced UC model in mice, Physalin B alleviated weight loss, improved clinical signs, and mitigated pathological damage. This effect is attributed to its ability to inhibit the activation of NF-κB, signal transducer and activator of transcription 3 (STAT3), β-arrestin1, and the NLRP3 inflammasome (Zhang Q. et al., 2020). Similarly, other steroids, such as Dioscin (Cai et al., 2021) has also been implicated in anti-inflammatory activities and may contribute to the therapeutic landscape for UC. Together, these findings highlight the importance of steroids such as Dioscin, Ruscogenin, and β-Sitosterol in treating UC, showing their diverse mechanisms of action involving both immune regulation and microbiota management.

### 5.7 Others

Figure 3G illustrates the currently reported other compounds that target pyroptosis for the treatment of UC. Diacetylrhein (DAR), an anti-inflammatory compound traditionally used for osteoarthritis, has demonstrated efficacy in targeting the NLRP3 inflammasome. Recent findings indicate that DAR inhibits inflammasome assembly and reduces caspase-1 activity, along with the inflammatory cytokines IL-1β and IL-18, thereby curbing the pyroptosis process. Additionally, DAR exhibits notable anti-apoptotic effects by modulating the balance of pro-apoptotic and anti-apoptotic proteins. It disrupts NF-κB signaling, leading to improved microscopic features in inflamed colon tissue and a reduced colon weight-to-length ratio, indicative of diminished disease activity and macroscopic damage. Furthermore, DAR is associated with decreased myeloperoxidase activity, IL-6, and TGF-β levels, as well as an increased expression of tight junction proteins, including occludin and ZO-1 (Zohny et al., 2022).

Salidroside, an active compound derived from *Rhodiola rosea* L., exhibits a spectrum of pharmacological activities, including anti-inflammatory, anti-cancer, and antioxidant properties (Xue et al., 2019). Recent research indicates that salidroside significantly slows the progression of UC by inhibiting pyroptosis in intestinal macrophages, enhancing the diversity of the intestinal microbial community, and modulating the Th17/Treg cell ratio (Liu X. et al., 2023). Furthermore, salidroside effectively alleviates dextran sulfate sodium (DSS)-induced colitis in murine models, primarily through the reduction of inflammation, the inhibition of pro-inflammatory pathways, the upregulation of PPARγ expression, and the restoration of intestinal barrier function (Liu et al., 2019).

Pectic polysaccharides (PPs) derived from *Rauwolfia verticillata* var. Hainanensis have also been shown to inhibit caspase-1 and IL-1β expression while promoting cellular pyroptosis, thereby impeding the progression of UC (Cui et al., 2022). Another study reported that PPs limit miR-124-3p and target RBP4, reducing the binding affinity of RBP4 to NLRP3. This inhibition of NLRP3-mediated pyroptosis leads to a decrease in pyroptosis of colonic epithelial cells and alleviates mucosal damage in UC (Yuan et al., 2023).

Trans-10-Hydroxy-2-Decenoic Acid (10-HDA) has been shown to effectively alleviate DSS-induced UC in mice by modulating the NLRP3 inflammasome-mediated pyroptotic pathway and enhancing colonic barrier function. Administration of 10-HDA significantly reduces pathological damage, ROS accumulation, neutrophil infiltration, and cytokine production in colonic tissues. It lowers the expression levels of several inflammatory markers, including TXNIP, NLRP3, ASC, caspase-1, GSDMD, IL-1β, and IL-18. Additionally, 10-HDA enhances colonic barrier integrity by increasing the expression of tight junction proteins ZO-1 and occludin. *In vitro* studies indicate that pretreatment with 10-HDA inhibits inflammasome-mediated pyroptosis in LPS/ATP-stimulated THP-1 cells, supporting its protective effects against colitis (Huang et al., 2022). In summary, various natural compounds like DAR, Salidroside, PPs, 10-HDA, and others have shown promising results in reducing UC by inhibiting NLRP3 inflammasome-mediated pyroptosis and inflammation. These compounds provide potential therapeutic strategies for treating UC by improving colonic barrier function, decreasing oxidative stress, and regulating inflammatory pathways.

### 5.8 Traditional Chinese medicine

TCM has been explored as a therapeutic option. Table 2 illustrates the currently reported TCM targeting pyroptosis for the treatment of UC, including formulas, extracts and other classes. Among various TCM formulations, the Xianglian pill (XLP) has gained attention for its effectiveness in UC treatment. Xianglian pill (XLP) is a TCM widely used in the treatment of UC (Zhang J. X. et al., 2024). Studies have reported that XLP could suppress myeloperoxidase (MPO) activity and decreased serum levels of inflammatory cytokines, including IL-1β, IL-6, TNF-α, and IL-18. Furthermore, XLP could inhibit the expression of key proteins associated with inflammation and apoptosis, such as GSDMD-N, TLR4, NLRP3, active-caspase-1, MyD88, and p-NF-κB/NF-κB in the colon tissues (Dai Y. et al., 2023). Another formulation, Kuijieling decoction (KJL), has been clinically utilized for several years, demonstrating significant therapeutic effects in UC management (Xiao et al., 2024). KJL treatment has been shown to markedly attenuate colonic injury while reducing inflammatory markers such as ASC, caspase-1, IL-1β, IL-18, NLRP3, and GSDMD-N in both *in vivo* and *in vitro* models of UC (Jie et al., 2021). Similarly, Xuanbi Yuyang decoction has been reported to ameliorate DSS-induced UC by inhibiting pyroptosis through blockade of IL-17 pathway activation (Huang X. et al., 2024). Kui Jie Tong (KJT) is recognized for its ability to restore body weight and colon length in DSS-induced rat models of UC. KJT significantly reduces Disease Activity Index (DAI) scores, alleviates colon damage, and inhibits key components of the NEK7-NLRP3/caspase-1 inflammatory pathway while also modulating intestinal microbiota. These findings underscore KJT’s potential to induce remission in UC (Xue et al., 2022).

**TABLE 2:** Targeting pyroptosis for the treatment of UC with TCM.

Types	Name	Dose	Cellular/animal models	Mechanisms	Ref.
Formulas	Xianglian Pill	0.45, 1.35, 2.7 mg/g	3% DSS-induced UC mice	By regulating the TLR4/MyD88/NF-κB signaling pathway	Dai et al. (2023a)
	Kuijieling decoction	*In vivo*: 4.6, 9.2, 18.3 mg/kg; *In vitro*: 2.5%, 5%, 10% KJL-containing serum	3% DSS-induced UC mice; LPS (100 ng/mL) and nigericin (10 μM)-induced RAW264.7 cells	By inhibiting activation of NLRP3 inflammasome	Jie et al. (2021)
	Xuanbi Yuyang Decoction	*In vivo*: 0.3, 0.4 mL; *In vitro*: XBD-containing serum	3% DSS-induced UC mice; 2% DSS-induced NCM460 cells	Via inhibition of IL-17 signaling pathway activation	Huang et al. (2024a)
	Kui jie tong	10 mL/kg	5% DSS-induced UC rat	By regulating gut microbiota and NLRP3/Caspase-1 classical pyroptosis signaling pathway	Xue et al. (2022)
	Shen-Ling-Bai-Zhu-San	*In vivo*: 1.183, 2.366, 4.732 g/kg; *In vitro*: concentrations of 0.313%, 0.625%, 1.25%	3% DSS-induced UC mice; DSS + LPS-induced MCME cells	By inhibiting caspase-1/Caspase-11-mediated pyroptosis	Chao et al. (2020)
	Huangqin Decoction	4.55, 9.1, 18.2 g/kg	3% DSS-induced UC mice	NLRP3/caspase-1 signaling pathway	Wu et al. (2021)
	Shenlingbaizhu formula	1.8, 3.6 g/kg; 3.6 g/kg SLBZ-containing serum	2% DSS-induced UC mice; Bone marrow-derived macrophage (BMDM); Intestinal organoid	By improving pathological symptoms, modulating gut microbiota and metabolic profiles, and inhibiting pro-inflammatory macrophage activation and TNFα-induced pyroptosis in intestinal organoids	Yu et al. (2022b)
	Tou Nong powder	3.3, 6.6, 13.2 g/kg	5% TNBS and 95% ethanol solution-induced UC rat	Through the regulation of NF-κB/NLRP3/Caspase-1/GSDMD inflammasome pyroptotic pathway	Ye et al. (2023)
	Huazhuojiedu decoction	10, 20, 40 g/kg	3.5% DSS-induced UC mice	By reducing inflammation, oxidative stress, and restraining the NLRP3/caspase-1 signaling pathway	Jia et al. (2022)
	Xiao-Jian-Zhong formula (XJZ)	*In vivo*: 5, 10.5 g/kg; *In vitro*: XJZ serum	2% DSS-induced UC mice; Macrophage (Mφ); Intestinal organoid	By resolving inflammation, restoring microbiome diversity, and promoting mucosal healing through mechanisms such as suppression of pyroptosis, enhancement of PPAR-γ expression, and inhibition of TNF-α-induced oxidative stress	Yu et al. (2022a)
	Yu Shi An Chang Fang	1, 10 mL/kg	100 mg/kg TNBS-induced UC mice	By Reducing Inflammatory Response and Protecting the Intestinal Mucosal Barrier	Ye and Lai (2021)
	Shaoyao decoction	*In vivo*: 1.12, 2.25 g/kg; *In vitro*: XJZ serum	5% DSS-induced UC mice; LPS-induced RAW 264.7 cells	Through the NLRP3, NF-κB P65 and P38 pathways	Wei et al. (2021)
	Jian-Wei-Yu-Yang Formula	*In vivo*: 0.7, 1.44 g/kg; *In vitro*: JW serum	2% DSS-induced UC mice; Macrophage (Mφ); Intestinal organoid	By inhibiting caspase-3-dependent pyroptosis, HIF-1α, and IL-1β, promoting alternative macrophage activation, and reducing TNF-α-induced ROS in intestinal organoids	Yan et al. (2022a)
	Wu-Mei-Wan	13.5, 27, 54 g/kg	2.5% DSS-induced UC mice	By inhibiting intestinal inflammatory response and repairing damaged intestinal mucosa	Yan et al. (2022b)
	Wuwei Kushen Changrong capsule	0.8256 g/kg; CSCC-containing serum	3% DSS-induced UC mice	Via inhibition of NLRP3 inflammasome and STAT3 pathway	Chen et al. (2024c)
	Tongxie-Yaofang formula	*In vivo*: 5.6, 11.2 g/kg; *In vitro*: 50, 100 mg/mL	3% DSS-induced UC mice; LPS/IFN-γ/ATP-stimulated BMDMs	Via NF-κB/NLRP3 signaling pathway	Zhang et al. (2022a)
	Qingre Xingyu recipe exerts	*In vivo*: 1.62 g crude drug/kg; *In vitro*: 10 μg/mL	3% DSS-induced UC mice; NLRP3^−/−^ Caco-2 cells; Peritoneal macrophages	By inhibiting TNFα/NLRP3/Caspase-1/IL-1β pathway and macrophage M1 polarization	Ning et al. (2023)
	Ganjiang Huangqin Huanglian Renshen Decoction	4, 8, 16 g/kg	3% DSS-induced UC mice	By attenuating inflammatory responses, inhibiting the TLR4/NF-κB/NLRP3 signalling, oxidative stress, and modulating the gut microbiota	Zhou et al. (2024a)
	Gegen Qinlian decoction	2.96, 11.83 g/kg	5% TNBS containing 50% ethanol-induced UC mice	By regulating Th2/Th1 and Tregs/Th17 cells balance, inhibiting NLRP3 inflammasome activation and reshaping gut microbiota	Hu et al. (2024)
	Qingchang Huashi Formula	0%, 50, 70, 90 ethanol extracts	2.5% DSS-induced UC mice	By restoring gut microbiota-metabolism homeostasis and goblet cell function	Hu et al. (2021)
	Modified Gegen Qinlian decoction	5, 10, 20 g/kg	2% DSS-induced UC mice	By restoring the intestinal mucus barrier and inhibiting the activation of γδT17 cells	Ma et al. (2023a)
Extracts	Astragalus mongholicus Bunge extract	250, 350, 450 mg/kg	3% DSS-induced UC mice	By enhancing PLCB2 expression and inhibiting colonic epithelial cell pyroptosis	Shen et al. (2024)
	*Forsythia suspensa* extract	0.1, 0.2, 0.4 g/mL	3% DSS-induced UC mice	Nrf2-NLRP3 Pathway	Chao et al. (2022)
	*Polygonum Hydropiper* L-*Coptis Chinensis*	114 mg/kg+ 228 mg/kg	3% DSS-induced UC mice	By regulating the NLRP3/Caspase-1 signaling pathway	Zhu et al. (2024)
	*Portulaca oleracea* L. aqueous extract (POE) and juice (PJ)	1 g/kg POE; 6, 12, 24 g/kg PJ	3% DSS-induced UC mice	By interfering with the activation of the NLRP3 inflammasome	Fan et al. (2023)
	*Puerariae radix*	0.068, 0.136, 0.272 mg/g	5% DSS-induced UC mice	By inhibiting NLRP3 inflammasome activation	Ga et al. (2024)
	*Patrinia villosa* Juss	21, 43, 64 g/kg	10 mg/kg TNBS and 0.25 mL 50% ethanol-water-induced UC rat	Via metabolism, vitamin D receptor and NF-κB signaling pathways	Wang et al. (2022a)
	*Agrimonia pilosa* Ledeb	3, 6 g/kg	3% DSS-induced UC mice	Through suppressing the activation of the NLRP3 inflammasome and NF-κB signaling pathways	Li et al. (2021)
	Rg3-enriched Korean Red Ginseng and Persicaria tinctoria extract	10 mg/kg Rg3-RGE +150 mg/kg PT	1.5% OXA-induced UC mice	Through the inhibition of the NF-κB pathway and reduced by NLRP3	Ullah et al. (2022)
	*Echinacea purpurea* (L.) Moench extract	1, 3 g/kg	60 mg/kg TNBS-induced UC rat	By inhibition of C3a/C3aR signaling pathway	Gu et al. (2023)
	*Canna x generalis* L.H. Bailey rhizome extract	100, 200 mg/kg	4% DSS-induced UC mice	Via modulating intestinal mucosal dysfunction, oxidative stress, inflammation, and TLR4/NF-ҡB and NLRP3 inflammasome pathways	Mahmoud et al. (2021)
	Hydroethanolic Extract of *Lepidium apetalum* Willdenow	*In vitro*: 12.5, 25, 50 μg/mL; *In vivo*: 100, 200 mg/kg	IL-6-stimulated Caco-2 cells; 5% DSS-induced UC mice	by enhancing intestinal barrier integrity and inhibiting oxidative stress and inflammasome activation	Kim et al. (2024)
	Tea (*Camellia sinensis*) water extracts	600 mg/kg	5% DSS-induced UC mice	By inhibiting TLR4/NF-κB/NLRP3 inflammasome	Liu et al. (2023c)
	Ethyl Acetate Extract from Decoction of *Sargentodoxa cuneata*	14.63, 29.25, 58.50 mg/kg	3% DSS-induced UC mice	By regulating TLR4/NF-κB/NLRP3 signaling pathway and decreasing the pro-inflammatory cytokine levels	Yu et al. (2023a)
	*Schisandra chinensis* (Turcz.) Baill	593.78, 1,187.55 mg/kg	3% DSS-induced UC mice	By regulating TLR4/NF-κB/NLRP3 inflammasome pathway and gut microbiota	Bian et al. (2022)
	*Forsythia suspensa* (Thunb.) Vahl extract	*In vivo*: 20, 60 mg/kg; *In vitro*: 25, 50, 100 μg/mL	2.5% TNBS-induced UC mice; LPS-induced PMA-stimulated THP-1 cells/BMDMs; Con A/LPS-induced lymphocytes abnormal proliferation; LPS/ATP-induced BMDM inflammation	Via inhibiting NLRP3 inflammasome activation through the TLR4/MyD88/NF-κB pathway	Tong et al. (2023)
	Polysaccharides from Garlic	150, 300 mg/kg	2.5% DSS-induced UC mice	Via suppressing pyroptosis and oxidative damage	Zhan et al. (2022b)
	Pectic polysaccharide from *Smilax chin*a L	*In vivo*: 250, 500 mg/kg; *In vitro*: 320, 640 μg/mL	3% DSS-induced UC mice; PMA-stimulated THP-1 cells	By inhibiting the galectin-3/NLRP3 inflammasome pathway	Pan et al. (2022b)
	Dandelion polysaccharide	200, 400 mg/kg	2% DSS-induced UC mice	By suppressing NF-κB/NLRP3 inflammasome-mediated inflammation and activating Nrf2	Wang et al. (2023b)
	Low Weight Polysaccharide of *Hericium erinaceus*	50, 100, 200 mg/kg	2% DSS-induced UC mice	Via inhibiting the NLRP3 inflammasome activation in association with gut microbiota modulation	Ren et al. (2023)
	Polysaccharides from *Hericium erinaceus*	200, 300, 400 mg/kg	3% DSS-induced UC mice	By inhibiting the NLRP3 inflammasomes and reestablish intestinal homeostasis	Li et al. (2024b)
	An inulin-type fructan CP-A from Codonopsis pilosula	*In vitro*: 50, 100 μg/mL; *In vivo*: 20, 40, 80 mg/kg	LPS-stimulated NCM460 cells; 3% DSS-induced UC mice	By promoting autophagy-mediated inactivation of NLRP3 inflammasome	Zhou et al. (2024b)
	*Codonopsis pilosula* polysaccharide	300, 600, 1,200 mg/kg	3% DSS-induced UC mice	By modulating gut microbiota and SCFA/GPR/NLRP3 pathway	Zhou et al. (2025)
Others	Walnut oil	100 mg/kg	3% DSS-induced UC mice	by inhibiting NLRP3 inflammasome activation and regulating gut microbiota	Miao et al. (2021)
	Yellow Teas	0.375 mg/kg	2% DSS-induced UC mice	By inhibiting TLR4/NF-κB/NLRP3 inflammasome	Xing et al. (2024)
	Honeysuckle	0.15, 0.75, 1.5 g/kg	2.5% DSS-induced UC mice	By reducing the production of pro-inflammatory cytokines, increases the content of short-chain fatty acids and restores the intestinal ecological balance	Zang et al. (2024)
	*Anastatica hierochuntica* essential oil	40, 80, 160 g/kg	2.5% DSS-induced UC mice	By regulating the NF-κB, PPARγ pathways, as well as the inhibition of NLRP3 activation	Alqudah et al. (2024)
	*Rubia cordifolia* L	250, 500 mg/kg	3% DSS-induced UC mice	Through dual inhibition of NLRP3 inflammasome and IL-6/JAK2/STAT3 pathways	Qin et al. (2022)
	*Platycodon grandiflorum* root fermentation broth	5 mL, 10 mL	3% DSS-induced UC mice	Through regulating AMPK/NF-κB/NLRP3 pathway	Wang et al. (2022d)

The Shen-ling-bai-zhu-san (SLBZS), referenced in the “Tai Ping Hui Min He Ji Ju Fang” has shown efficacy against various gastrointestinal disorders. Recent studies reveal that SLBZS reduces pro-inflammatory factor production, regulates MAPK and NF-κB signaling pathways, and inhibits pyroptosis signaling. Furthermore, SLBZS restores the expression of colonic tight junction proteins, thereby preserving the integrity of the colonic mucosal barrier (Chao et al., 2020). Huangqin decoction has demonstrated the ability to inhibit pyroptosis in UC cells, likely through the NLRP3/caspase-1 signaling pathway (Wu et al., 2021). The Shenlingbaizhu formula (SLBZ) also effectively alleviates DSS-induced UC by improving pathological symptoms, modulating gut microbiota and metabolic profiles, and inhibiting pro-inflammatory macrophage activation (Yu et al., 2022b). Tou Nong powder (TNP) has been shown to ameliorate UC by improving the DAI, reversing colonic weight loss, and alleviating histopathological injury. TNP treatment decreases levels of inflammatory markers, including TNF-α, diamine oxidase, ICAM-1, and endotoxin, while inhibiting the NF-κB/NLRP3/Caspase-1/GSDMD signaling pathway (Ye et al., 2023).

Huazhuojiedu decoction (HZJDD) has been clinically validated for UC treatment, with research indicating its role in regulating oxidative stress and downregulating key inflammatory markers such as CRP, TNF-α, IL-6, LPS, IL-1β, and IL-18. HZJDD also inhibits the activation of the NLRP3/caspase-1 signaling pathway, mitigating cellular death and enhancing therapeutic effects (Jia et al., 2022). The Xiao-Jian-Zhong formula (XJZ) treatment has shown promise in restoring gut microbial diversity and abundance, inhibiting pathogenic microbes, and enhancing linoleic acid metabolism and cytochrome P450 activity. *In vitro* experiments suggest that XJZ inhibits caspase-1-dependent pyroptosis, increases PPAR-γ expression, and promotes macrophage activation (Yu et al., 2022a). Yu Shi An Chang Fang (YST) has been reported to reduce levels of NLRP3, ASC, and cleaved caspase-1, while increasing the expression of tight junction proteins in colonic tissues. This suggests that YST ameliorates TNBS-induced UC by diminishing inflammatory responses and enhancing intestinal barrier function (Ye and Lai, 2021). Shao Yao Decoction (SYD), with historical significance dating back to the Jin-Yuan period, has demonstrated efficacy in UC treatment. SYD appears to protect against UC potentially by inhibiting the MKP1/NF-κB/NLRP3 pathway, thus alleviating cellular pyroptosis (Wei et al., 2021).

In addition to traditional compounded preparations, various herbal extracts have garnered considerable attention for their therapeutic potential in treating UC. Among these, Gegen-Qinlian decoction, which prominently features *Puerariae Radix*, has been utilized to alleviate intestinal pain and inflammation. Recent studies indicate that *Puerariae Radix* may ameliorate pyroptosis in DSS-induced UC models, exerting anti-inflammatory effects through the modulation of the NLRP3/caspase-1 signaling pathway (Ga et al., 2024). Similarly, a combination of *Polygonum Hydropiper* L. and *Coptis Chinensis* (PH-CC) has been demonstrated to inhibit pyroptosis-associated proteins and downregulate pro-inflammatory cytokines, including IL-18 and IL-1β. These findings support the notion that PH-CC may improve UC symptoms by regulating the NLRP3/caspase-1 pathway, underscoring its potential as a therapeutic agent for inflammatory bowel diseases (Zhu et al., 2024). Additionally, extracts of *Astragalus mongholicus* Bunge exhibit a spectrum of pharmacological effects, including anti-inflammatory, anticancer, and immunomodulatory properties. Studies reveal that these extracts can alleviate UC in DSS-induced mice by enhancing PLCB2 expression and inhibiting colonic epithelial cell pyroptosis, resulting in reduced inflammation and improved clinical and histopathological outcomes (Shen et al., 2024). *Forsythia suspensa* extract has also shown promise, significantly alleviating DSS-induced UC in mice by enhancing antioxidant activity and inhibiting pyroptosis through the Nrf2-NLRP3 signaling pathway, while concurrently improving metabolic function (Chao et al., 2022). *Portulaca oleracea* L., an edible and medicinal plant, has been recognized for its potential in treating gastrointestinal disorders. Comparative studies indicate that *P. oleracea* juice (PJ) contains a higher concentration of bioactive compounds and demonstrates a greater number of overlapping targets with UC compared to Portulaca oleracea extract (POE). In mouse models of UC, both POE and PJ were effective in reducing DAI scores and inflammatory cell infiltration; however, PJ exhibited superior efficacy. Furthermore, PJ inhibited cellular pyroptosis by downregulating the expression of NLRP3 inflammasome components and helped restore intestinal barrier function by upregulating tight junction proteins (Fan et al., 2023). In one study, polysaccharides derived from garlic (PSG) significantly alleviated inflammatory bowel disease and secondary liver injury induced by a drug delivery system (DDS) in mice. This effect was achieved by reducing colonic inflammation and enhancing intestinal barrier integrity. Mechanistically, PSG was found to suppress key inflammatory markers, including IL-1β, IL-18, and TLR4, alongside pyroptosis-related proteins such as NLRP3, GSDMD, and caspase-1, while also mitigating oxidative damage in liver tissues. These findings highlight the potential of PSG as a viable treatment for inflammatory bowel disease and secondary liver injury in humans (Zhan et al., 2022b). Overall, these studies collectively highlight the therapeutic promise of TCM, including compounds and extracts, in controlling UC by regulating the pathways associated with pyroptosis, which warrants further research into its mechanism of action and clinical application. The more detailed information of TCM formulas, extracts and other targeted pyroptosis treatments for UC are shown in Table 2.

## 6 Therapeutic potential and challenges

### 6.1 Therapeutic potential

The therapeutic spectrum for treatment of UC has expanded dramatically over the past few decades, with natural products emerging as potential adjunctive or alternative therapies to conventional therapies (Zheng et al., 2022; Xue et al., 2023). A large number of clinical trials have confirmed the efficacy of natural products in improving the symptoms of UC, which are mainly manifested in enhancing intestinal barrier function, reducing inflammatory response and improving the overall quality of life of patients (Guo et al., 2017; Zeng et al., 2022). For instance, SLBZ and Shaoyao decoction have been approved by the State Medical Products Administration in China for the treatment of UC and have shown promising therapeutic effects (Zhao Z. H. et al., 2024). In addition, other TCM compounds such as Curcumin have also been shown in clinical studies to restore the balance of intestinal microbiota and regulate NF-κB signaling in patients (Wang et al., 2018; Lin et al., 2022). When comparing natural products/TCM with conventional therapies, evidence suggests that while aminosalicylates, corticosteroids, and biologics offer robust short-term efficacy, their long-term use is limited by immunosuppression risks and relapse rates (Liu et al., 2022). The integration of natural products into treatment regimens could potentially mitigate some of these adverse effects while enhancing therapeutic outcomes. For example, a retrospective multicenter cohort study involving 88 patients with active UC (50% previously exposed to biologics/small molecule agents) demonstrated that the Curcumin-QingDai combination effectively induced clinical remission (46.5%) and biomarker response (median fecal calprotectin decreased from 1,000 μg/g to 75 μg/g), with no significant safety concerns observed. In conclusion, the current study suggests that the natural products/TCM preparations including SLBZ, Shaoyao decoction, and curcumin have good therapeutic potential in treating UC. Nevertheless, there is a need for further clarification of standardized protocols for the use of these natural products and TCM, optimization of dosage strategies and elucidation of their long-term therapeutic efficacy in different UC patient populations in the future.

### 6.2 Challenges and limitations

Natural products have shown significant potential in treating UC, but still face many challenges in their clinical application. Key issues include bioavailability, standardisation and potential interactions with conventional drugs. (1) Firstly, bioavailability is one of the most critical issues because many natural compounds (e.g., Quercetin) are characterized by poor absorption and rapid metabolism, which may reduce their therapeutic efficacy considerably. Whereas, current reports show that strategies such as nanoparticle formulations and liposomal drug delivery show great promise for improving the bioavailability of Quercetin (Tomou et al., 2023). Therefore, the potential for clinical development of natural products can be greatly enhanced by strategies such as nanoparticle formulation and liposomal drug delivery. (2) Secondly, the standardization and quality control of natural products are also challenges that hinder their further application in the field of UC. The content or clinical efficacy of these natural products varies greatly depending on the geographical location of the plant from which they are derived and the processing method of the part of the plant where they are used. To address this, establishing stringent quality control measures and standardized extraction protocols is vital for ensuring the safety and efficacy of natural products in UC treatment. (3) Moreover, the potential for adverse effects and drug interactions must be carefully considered. While often perceived as safe, natural products can cause adverse reactions or interact with prescribed medications, particularly through modulation of cytochrome P450 enzymes (Jackson et al., 2018). In short, while natural products offer valuable therapeutic options for UC, challenges related to bioavailability, standardization and drug interactions must be addressed for safe and effective clinical application.

## 7 Conclusion and future directions

**FIGURE 4:**
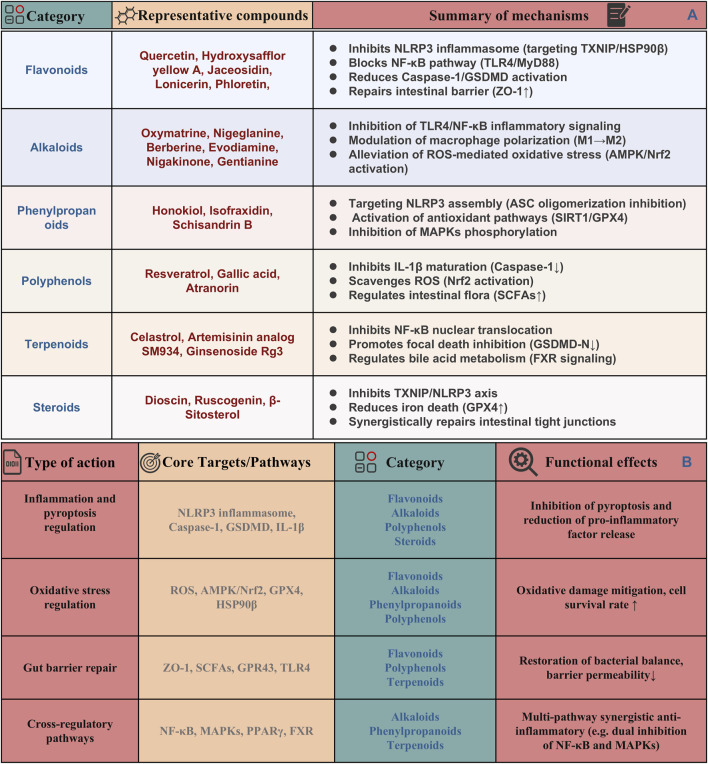
Categorization and summary of natural products types, functions, core targets and pathways by targeting pyroptosis for UC treatment. **(A)** Potential regulatory targets and mechanisms of natural products targeting pyroptosis for the treatment of UC. **(B)** Integrative network of compound-target/pathway interactions across inflammation, oxidative stress, and gut barrier repair.

Natural products present a promising approach for treating UC, offering diverse bioactive compounds with anti-inflammatory and cytoprotective properties. For instance, flavonoids, polyphenols, and terpenoids have demonstrated efficacy in attenuating inflammatory responses through the inhibition of pyroptotic signaling cascades, thereby reducing mucosal damage and fostering intestinal healing (Wang T. et al., 2024). As the understanding of pyroptosis and its implications in UC deepens, the potential for natural products to serve as adjuncts or even primary treatments becomes increasingly evident. In this review, we find that specific phytochemical subclasses demonstrate multi-target interventions against UC-associated pyroptosis (Figure 4B). Notably, selected flavonoids (e.g., Phloretin, Naringin, and Rutin) and alkaloids (e.g., Oxymatrine and Evodiamine) suppress NLRP3 inflammasome activation mediated pyroptosis signaling, while steroids like β-sitosterol inhibit Caspase-1/GSDMD-mediated pyroptotic signaling. Concurrently, certain phenylpropanoids (e.g., Schisandrin B) and terpenoids (e.g., Brusatol) enhance redox homeostasis by scavenging ROS via Nrf2 pathways and activating AMPK-dependent stress adaptation, indirectly attenuating oxidative stress-triggered pyroptosis. Gut barrier restoration is achieved through SCFAs-GPR43/TLR4 axis modulation by terpenoids (e.g., *P. chinensis* saponins) which rebalance microbiota and fortify epithelial integrity to prevent pyroptosis-inducing pathogen infiltration. Cross-regulatory mechanisms further amplify anti-pyroptotic effects via dual inhibition of NF-κB/MAPKs cascades (e.g., *Platycodon grandiflorum* root) and PPARγ/FXR-mediated transcriptional control (e.g., Nigakinone, Phloretin), creating synergistic networks that disrupt chronic inflammatory amplification loops. All in all, the structural diversity of phytochemicals enables polypharmacological targeting of these interconnected axes, highlighting their therapeutic superiority over single-pathway agents in addressing UC’s multifactorial pyroptosis pathology.

In the future, it is need to prioritize continued exploration into the mechanisms by which natural products influence pyroptosis and related inflammatory pathways. Research should focus on elucidating the molecular interactions of these natural compounds with the key components in cell pyroptosis. Additionally, it is need to develop standardized formulations or extracts to ensure consistency in therapeutic efficacy and safety in clinical. Alongside basic research, clinical investigations are equally vital for translating these findings into practical applications. Rigorous clinical trials are necessary to evaluate the effectiveness and safety of interventions using natural products in patients with UC, confirming their role in clinical management. Moreover, studying the pharmacokinetics and bioavailability of these compounds will be crucial for understanding their therapeutic windows and potential interactions with existing drugs. Furthermore, interdisciplinary approaches that combine insights from microbiology (Shen et al., 2018; Kang et al., 2023), immunology (Long et al., 2024b), and pharmacology (Wang Z. et al., 2024) will enhance our understanding of the complex interactions between pyroptosis, inflammation, and the gut microbiome in UC. The gut microbiome plays a pivotal role in modulating immune responses, and its dysbiosis has been implicated in the pathogenesis of UC. Understanding how pyroptosis influences microbial composition and function, as well as how natural products can restore microbial balance, could lead to innovative strategies for UC management.

Emerging technologies, such as metagenomics and metabolomics, offer powerful tools to explore these interactions in greater detail (Li M. et al., 2022; Schirmer et al., 2024). By characterizing the microbial communities in UC patients and assessing the impact of natural products on these communities, it is possible to inform personalized medicine for UC patients. Moreover, the role of lifestyle factors (Lo et al., 2021; Lopes et al., 2022), such as diet and physical activity, in modulating pyroptosis and inflammation in UC deserves further investigation. Dietary interventions that incorporate natural products rich in anti-inflammatory constituents may synergize with pharmacological therapies to enhance patient outcomes. A holistic approach that combines dietary modifications with natural product supplementation could provide a comprehensive strategy for managing UC and improving quality of life for patients.

In conclusion, the significance of pyroptosis in UC cannot be overstated. Its role in driving inflammation and tissue damage presents both challenges and opportunities for therapeutic intervention. Natural products represent a promising frontier in the exploration of new treatments for UC, with the potential to modulate pyroptosis and restore intestinal homeostasis. Continued research into the mechanisms underlying these interactions and the clinical applicability of natural products will be vital in shaping the future landscape of UC management. By embracing a multifaceted approach that integrates basic and clinical research, we can unlock the full potential of natural products as therapeutic agents in the fight against UC.

## Glossary

AIM2: absent in melanoma 2
APE: Apple polyphenols extract
BBR: Berberine
CSR: celastrol
COX2: cyclooxygenase-2
CAT: catalase
DSS: dextran sulfate sodium
DAR: Diacetylrhein
DAI: Disease Activity Index
DAMPs: damage-associated molecular patterns
EVO: Evodiamine
GA: Gallic acid
GSDMD: gasdermin D
GSDME: gasdermin E
GTN: Gentianine
GSH: glutathione
HSP-90: heat shock protein 90
HZJDD: Huazhuojiedu decoction
KJT: Kui Jie Tong
MPO: myeloperoxidase
MDA: malondialdehyde
MPO: myeloperoxidase
NOS: nitric oxide synthase
NRF2: nuclear factor erythroid 2-related factor 2
NLRs: NOD-like receptors
PAMPs: pathogen-associated molecular patterns
PRRs: pattern recognition receptors
Pg3gal: Pelargonidin-3-galactoside
PPs: Pectic polysaccharides
PH-CC: Polygonum Hydropiper L. and Coptis Chinensis
PJ: Portulaca oleracea juice
POE: Portulaca oleracea extract
PSG: polysaccharides derived from garlic
SOD1: superoxide dismutase 1
SCFAs: short-chain fatty acids
STAT3: signal transducer and activator of transcription 3
SM934: β-aminoarteether maleate
SLBZS: Shen-ling-bai-zhu-san
SLBZ: Shenlingbaizhu formula
SYD: Shao Yao Decoction
TLR4: Toll-like receptor 4
TCM: Traditional Chinese medicine
TNP: Tou Nong powder
UC: Ulcerative colitis
XLP: Xianglian pill
XJZ: Xiao-Jian-Zhong formula
YST: Yu Shi An Chang Fang
ZO-1: zonula occludens-1
10-HAD: Trans-10-Hydroxy-2-Decenoic Acid
